# Factors Associated with Ventriculoperitoneal Shunt Placement Following Posterior Fossa Tumor Resection in Children: A Systematic Review and Meta-Analysis

**DOI:** 10.3390/medicina62101953

**Published:** 2026-10-09

**Authors:** Younis Al-Mufargi, Asma AlHosni, Ayat Al Zadjali, Manar Al-Farsi, Al-Zahraa Al-Arafati, Said Al-Mayahi, Mohammed Al Subhi, Tariq Al-Saadi

**Affiliations:** 1 Department of General Surgery, Medical City for Military and Security Services, Muscat 121, Oman; 2 Department of Neurosurgery, Gui de Chauliac Hospital, CHU de Montpellier, University of Montpellier, 80 Avenue Augustin Fliche, 34295 Montpellier, France; namesss941@gmail.com; 3 Department of Paediatrics, Al Rustaq Hospital, Rustaq 329, Oman; ayat.alz44@gmail.com; 4 Department of General Surgery, Sohar Hospital, Sohar 311, Oman; manoor1611@gmail.com; 5 College of Medicine and Health Sciences, Sultan Qaboos University, Muscat 123, Oman; arafzah9@gmail.com; 6 Department of Neurosurgery, Khoula Hospital, Muscat 116, Oman; almayahisaid2707@gmail.com; 7 School of Medicine, Medical Sciences and Nutrition, University of Aberdeen, Foresterhill, Aberdeen AB25 2ZD, UK; m.alsubhi.23@abdn.ac.uk; 8 Department of Neurosurgery, Cedars-Sinai Medical Center, 8700 Beverly Blvd, Los Angeles, CA 90048, USA

**Keywords:** posterior fossa tumor, ventriculoperitoneal shunt, pediatric neurosurgery, hydrocephalus, cerebrospinal fluid, associated factors, meta-analysis

## Abstract

*Background and Objectives*: Posterior fossa tumors (PFTs) are common pediatric brain tumors and frequently present with obstructive hydrocephalus. Although tumor resection restores cerebrospinal fluid (CSF) circulation in many patients, a subset subsequently requires ventriculoperitoneal shunt (VPS) placement. This systematic review and meta-analysis aimed to identify factors associated with postoperative VP shunt placement following PFT resection in children. *Materials and Methods*: This systematic review and meta-analysis was conducted in accordance with the Preferred Reporting Items for Systematic Reviews and Meta-Analyses (PRISMA) guidelines and was prospectively registered with PROSPERO (CRD420261455396). A comprehensive search of major electronic databases was conducted from inception to April 2025. Observational studies evaluating factors associated with postoperative VP Shunt placement in children undergoing PFT resection were included. Risk of bias was assessed using the Quality In Prognosis Studies (QUIPS) tool, and certainty of evidence was evaluated using the modified Grading of Recommendations Assessment, Development and Evaluation (GRADE) framework for prognostic factor research. Pooled odds ratios (ORs) with 95% confidence intervals (CIs) were calculated using random-effects meta-analysis. *Results*: Nine retrospective cohort studies comprising 922 children were included. Higher odds of postoperative VP shunt placement were observed with preoperative hydrocephalus (OR = 7.62, 95% CI 2.19–26.47), midline tumor location (OR = 4.75, 95% CI 1.21–18.69), ependymoma (OR = 2.56, 95% CI 1.38–4.76), subtotal resection (OR = 3.04, 95% CI 1.92–4.79), postoperative intraventricular hemorrhage (OR = 4.36, 95% CI 1.16–16.44), postoperative infection (OR = 4.96, 95% CI 1.77–13.91), and pseudomeningocele (OR = 6.17, 95% CI 3.12–12.19). Astrocytoma was associated with lower odds of VP shunt placement (OR = 0.47, 95% CI 0.27–0.85), whereas sex, medulloblastoma, and postoperative CSF leakage were not significantly associated. EVD exposure was associated with higher odds of subsequent VP shunt placement (OR = 4.79, 95% CI 2.11–10.87), although this association is susceptible to confounding by indication. Certainty of evidence ranged from very low to low, with most pooled estimates derived from unadjusted data. *Conclusions*: Several clinical and perioperative characteristics may help identify children requiring closer surveillance for persistent hydrocephalus after posterior fossa tumor resection. The observed relationship with EVD likely reflects underlying hydrocephalus severity rather than an independent prognostic effect. Given the limited certainty and predominantly unadjusted evidence, these findings should support risk-informed surveillance rather than decisions for permanent CSF diversion. Future prospective studies with standardized definitions and multivariable adjustment are needed to develop reliable prediction models.

## 1. Introduction

The posterior fossa is an intracranial compartment that contains the brainstem and cerebellum. If an obstruction to cerebrospinal fluid (CSF) pathways develops in this region, it can hinder the flow of cerebrospinal fluid that is circulating around the brain and spinal cord [1,2,3]. This can lead to increased intracranial pressure and hydrocephalus [1,2,4].

Posterior fossa tumors in children are often primary and typically arise sporadically; however, certain types, such as medulloblastomas, may be associated with genetic syndromes including Li-Fraumeni, Gorlin, and Turcot syndromes [1,2,5,6]. Due to their location within a confined space, these tumors can result in life-threatening complications such as brainstem compression, tonsillar herniation, and raised intracranial pressure [7,8]. Symptoms often present early and include headache, imbalance, nausea, vomiting, and gait disturbance. Posterior fossa tumors are a common cause of hydrocephalus in children, which can complicate tumor resection performed to relieve mass effect and intracranial pressure [2,5].

In some cases, tumor resection alone is insufficient, and a subset of patients will have persistent hydrocephalus postoperatively [9,10]. Therefore, CSF diversion procedures, such as ventriculoperitoneal (VP) shunting, play a crucial role in management [9,10]. These procedures divert excess CSF to another body cavity, most commonly the peritoneal cavity. However, they are associated with risks including infection, hemorrhage, obstruction, and shunt malfunction [11].

Previous studies have evaluated demographic, clinical, radiological, tumor-related, operative, and postoperative factors in relation to VP shunt placement after pediatric PFT resection. However, the available evidence is predominantly retrospective, definitions of hydrocephalus and indications for permanent CSF diversion vary across institutions, and the extent of adjustment for potential confounding varies substantially across studies. Moreover, therapeutic interventions such as external ventricular drainage (EVD) may reflect underlying hydrocephalus severity and treatment selection rather than independent prognostic effects. These limitations complicate interpretation of individual studies and highlight the need for a systematic synthesis of the available evidence.

Therefore, this systematic review and meta-analysis aimed to synthesize the available evidence on factors associated with postoperative VP shunt placement following PFT resection in children, evaluate the certainty of these associations, and identify limitations in the current evidence to inform future prognostic research.

## 2. Materials and Methods

### 2.1. Study Design and Literature Search

This systematic review and meta-analysis was conducted in accordance with the Preferred Reporting Items for Systematic Reviews and Meta-Analyses (PRISMA) 2020 guidelines (File S1). The review protocol was prospectively registered with the International Prospective Register of Systematic Reviews (PROSPERO registration Number: CRD420261455396). A comprehensive literature search was performed to identify studies reporting factors associated with VP shunt placement following posterior fossa tumour (PFT) resection in paediatric patients. The search was conducted across six electronic databases: PubMed/MEDLINE, Scopus, Wiley Online Library, Web of Science, EBSCOhost, and Google Scholar. The search strategy combined Medical Subject Headings (MeSH), where applicable, and free-text keywords using Boolean operators, including “*posterior fossa tumour*” OR “*cerebellar tumour*” OR “*brain tumour*” AND “*ventriculoperitoneal shunt*” OR “*VPS*” OR “*hydrocephalus*” AND “*predictors*” OR “*risk factors*” OR “*determinants*”. The search was limited to articles published in English involving human paediatric subjects (<18 years of age). In addition, the reference lists of all eligible studies and relevant review articles were manually screened to identify any additional publications that met the inclusion criteria.

### 2.2. Eligibility Criteria and Study Selection

**Figure 1:**
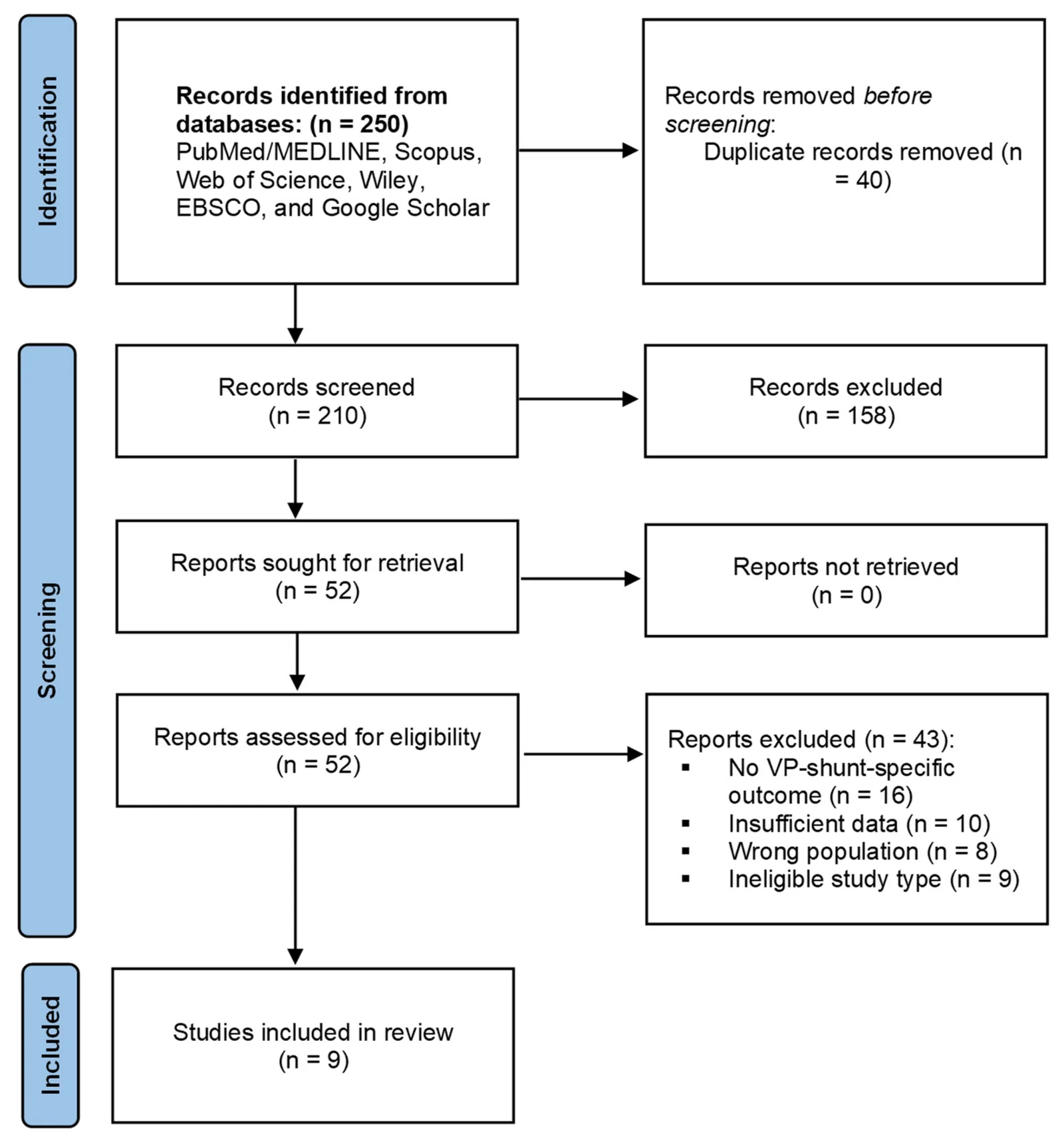
PRISMA flow diagram of study selection for systematic review and meta-analysis on associated factors of VP shunt placement in pediatric posterior fossa tumors.

Original observational studies evaluating factors associated with postoperative VP shunt placement in children (<18 years of age) undergoing posterior fossa tumor resection were considered eligible. Studies were required to report sufficient data to estimate the association between at least one risk factor and postoperative VP shunt placement. Review articles, case reports, conference abstracts, editorials, studies involving adult populations, and studies lacking extractable outcome data were excluded. Two reviewers independently screened titles and abstracts, followed by full-text assessment of potentially eligible studies. Disagreements were resolved through discussion and consensus. Potential cohort overlap was assessed by cross-checking study institution, recruitment period, patient population, sample size, tumor characteristics, and authorship. Studies from the same or potentially related centers with overlapping study periods were further compared for cohort characteristics and eligibility criteria. No duplicate or substantially overlapping patient populations were identified, and all included studies were considered independent cohorts. The study selection process is summarized in PRISMA flow chart (Figure 1).

### 2.3. Data Extraction and Outcomes Measures

Two reviewers independently extracted data using a standardized data collection form. Extracted information included study characteristics (author, publication year, country, study period, design, and sample size), patient demographics, tumor characteristics, preoperative hydrocephalus, CSF-diversion procedures, operative and postoperative factors, and postoperative VP shunt placement. Where available, definitions and assessment methods for hydrocephalus, quantitative ventricular indices, indications and timing of EVD and VP shunt placement, follow-up duration, and VP shunt incidence were also extracted. For each study, we recorded whether associations were evaluated using univariable or multivariable methods and extracted reported adjusted estimates and covariates where available. Crude and adjusted effect estimates were distinguished during data extraction. Where multivariable or confounder-adjusted analyses were reported, the statistical model, covariates included or controlled, and principal adjusted findings were extracted separately and summarized in Supplementary Table S1**.** Evaluated variables were categorized as demographic, tumor-related, preoperative, operative, or postoperative factors. EVD exposure was analyzed separately as a therapeutic intervention and marker of hydrocephalus severity rather than classified as an independent prognostic factor. EVD was retained in the quantitative synthesis because it represented a clinically relevant management exposure evaluated in multiple eligible studies in relation to subsequent VP shunt placement. However, unlike intrinsic patient- or tumor-related characteristics, EVD placement is influenced by clinical status, hydrocephalus severity, and treatment selection. EVD was therefore analyzed as a therapeutic/management exposure rather than as an independent prognostic factor, and its association with subsequent VP shunt placement was considered particularly susceptible to confounding by indication. Outcome definitions were harmonized by considering subsequent VP shunt placement following PFT resection as the outcome of interest. Where cohorts included patients who had undergone permanent VP shunting as part of initial hydrocephalus management, these patients were not considered incident postoperative shunt events. Specifically, in Dias and Albright, analyses of subsequent VP shunt placement were based on the initially non-shunted subgroup. The primary outcome was postoperative VP shunt placement following PFT resection.

### 2.4. Risk-of-Bias Assessment

The methodological quality of the included studies was independently assessed by two reviewers using the Quality In Prognosis Studies (QUIPS) tool [12]. Six domains were evaluated: study participation, study attrition, prognostic factor measurement, outcome measurement, confounding, and statistical analysis and reporting. Disagreements were resolved by consensus. Domain-level judgments for each included study are provided in Supplementary Table S3.

### 2.5. Certainty of Evidence

The certainty of evidence for each factor or exposure associated with postoperative VP shunt placement was assessed using the modified Grading of Recommendations Assessment, Development and Evaluation (GRADE) framework for prognostic factor research proposed by Huguet et al. [13]. The initial certainty rating was determined according to the phase of prognostic investigation. Because the available evidence predominantly comprised retrospective exploratory studies with variable adjustment for potential confounding and limited evidence establishing independent prognostic effects, the evidence was considered predominantly phase 1 and therefore started at moderate certainty. Certainty was subsequently assessed across the domains of study limitations, inconsistency, indirectness, imprecision, and publication bias. Evidence was considered for upgrading when a moderate or large magnitude of association or an exposure–response gradient was demonstrated. However, because the pooled estimates were derived from unadjusted event data, effect magnitude alone was not considered sufficient for upgrading when important residual confounding could plausibly contribute to the observed association. This consideration was particularly relevant to EVD, which was treated as a management exposure susceptible to confounding by indication, and to postoperative factors for which temporality relative to VP shunt placement was uncertain. Risk-of-bias judgments from the Quality In Prognosis Studies (QUIPS) assessment informed the study-limitations domain. The availability and adequacy of confounder adjustment were also considered within this domain. Because comparable adjusted estimates were insufficient for quantitative pooling, the potential for residual confounding was considered when assigning factor-specific certainty ratings, particularly where contributing studies had substantial confounding concerns. The overall certainty of evidence for each factor was categorized as high, moderate, low, or very low.

### 2.6. Statistical Analysis

Meta-analyses were performed using a random-effects model to account for anticipated clinical and methodological heterogeneity across studies. Pooled odds ratios (ORs) with corresponding 95% confidence intervals (CIs) were calculated for factors or exposures reported by at least two studies. Statistical heterogeneity was assessed using Cochran’s *Q* test and quantified using the *I*^2^ statistic. For postoperative infection, meningitis and CSF infection were analyzed separately as subgroups, with an overall pooled infection estimate additionally calculated; differences between subgroup estimates were assessed using a test for subgroup differences. Leave-one-out sensitivity analyses were performed to assess the influence of individual studies on pooled estimates, particularly for outcomes demonstrating substantial heterogeneity. To assess the influence of historical differences in neurosurgical and CSF-diversion practices, factor-specific sensitivity analyses were performed after excluding contributing historical cohorts published between 1989 and 1996, where sufficient studies remained for quantitative synthesis. Specifically, Culley et al. (1994) was excluded from the tumor-location analysis [14]; Dias and Albright (1989) and Lee et al. (1994) from the EVD analysis [15,16]; Kumar et al. (1996) from the medulloblastoma, astrocytoma, and ependymoma analyses [6]; and Kumar et al. (1996) and Lee et al. (1994) from the extent-of-resection analysis [6,16]. Historical cohorts contributing to the postoperative infection analysis were similarly excluded. The resulting estimates were compared with the primary analyses to assess the robustness of the pooled associations. Statistical significance was defined as a two-sided *p* < 0.05. Forest plots were generated to summarize pooled effect estimates for demographic, tumor-related, preoperative, operative, and postoperative factors, as well as EVD exposure, associated with subsequent VP shunt placement. Publication bias and small-study effects were not formally assessed using funnel plots or statistical tests for funnel-plot asymmetry because fewer than 10 studies contributed to each factor-specific meta-analysis. Under these circumstances, such methods have limited statistical power and may yield unreliable assessments of asymmetry [17]. All analyses were conducted through R version 4.5.1 (R Foundation for Statistical Computing, Vienna, Austria) using the *meta* package.

## 3. Results

### 3.1. Study Selection

The literature search identified 250 records from PubMed/MEDLINE, Scopus, Web of Science, Wiley Online Library, EBSCOhost, and Google Scholar. After removal of 40 duplicate records, 210 unique studies remained for title and abstract screening. Following initial screening, 52 full-text articles were assessed for eligibility. Of these, 43 studies were excluded because they did not report ventriculoperitoneal (VP) shunt-specific outcomes, included adult populations, provided insufficient data for meta-analysis, or were review articles or case reports. Ultimately, nine retrospective cohort studies met the eligibility criteria and were included in both the qualitative systematic review and quantitative meta-analysis (Figure 1).

### 3.2. Study Characteristics

The characteristics of the included studies are summarized in Table 1. The nine retrospective cohort studies were published between 1989 and 2023 and collectively included 922 pediatric patients who underwent posterior fossa tumor resection. Sample sizes ranged from 26 to 217 patients. The studies were conducted across India (*n* = 2), the United States (*n* = 3), the United Kingdom (*n* = 1), Germany (*n* = 1), China (*n* = 1), and a multicenter cohort from the United Kingdom and Hong Kong (*n* = 1). The mean or median patient age ranged from 5.5 to 9.3 years, where reported. Among studies reporting sex distribution, males accounted for approximately 56–67% of participants. Most studies included heterogeneous posterior fossa tumor populations, whereas Afshari et al. and Lee et al. specifically evaluated children with medulloblastoma. All included studies evaluated postoperative VP shunt placement as the primary outcome. Variables evaluated across studies encompassed demographic characteristics, tumor-related factors, preoperative clinical and radiological factors, operative factors, postoperative complications, and CSF-diversion management exposures. These included patient sex, tumor location and histology, preoperative hydrocephalus, extent of tumor resection, postoperative intraventricular hemorrhage, CSF leakage, postoperative infection, and pseudomeningocele. EVD was evaluated separately as a clinically relevant management exposure associated with subsequent VP shunt placement. Additional study-level information regarding analytical approaches and covariate adjustment is provided in Supplementary Table S1, while definitions and assessment of hydrocephalus and CSF-diversion practices are summarized in Supplementary Table S2.

Four of the nine included studies (44.4%) reported formal multivariable analyses, two (22.2%) used partial or stratified adjustment, and three (33.3%) reported only unadjusted analyses (Supplementary Table S1). However, comparable adjusted estimates were insufficient for factor-specific quantitative pooling. Consequently, all 12 primary factor-specific meta-analyses were derived from crude/unadjusted study-level event data (12/12, 100%), and none were based exclusively on adjusted effect estimates (0/12, 0%). The pooled ORs therefore represent crude associations rather than independent prognostic effects.

### 3.3. Risk of Bias Assessment

**Figure 2:**
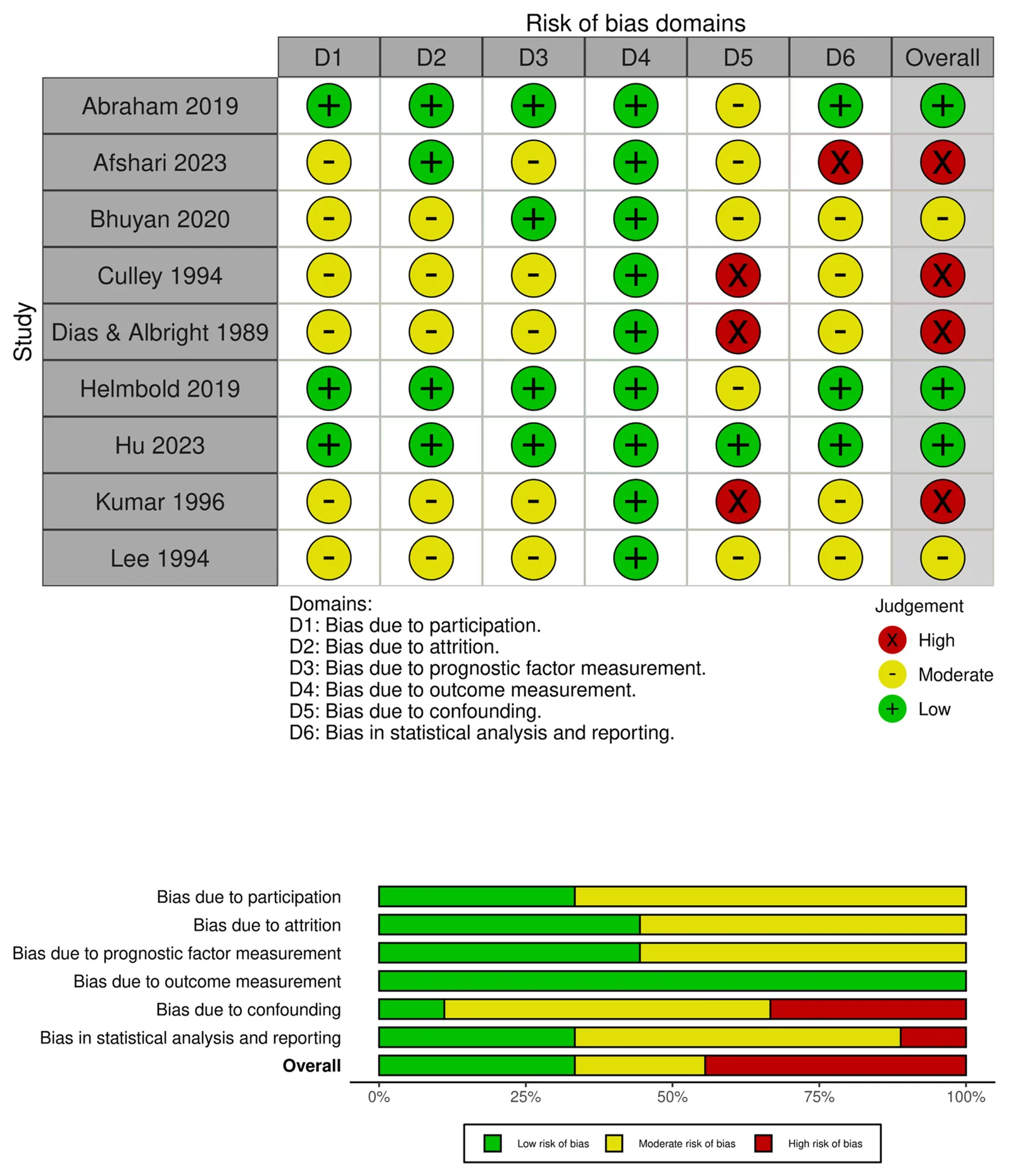
Risk-of-bias assessment of the included studies using the Quality In Prognosis Studies (QUIPS) tool [6,14,15,16,18,19,20,21,22].

The methodological quality of the included studies was assessed using the Quality In Prognosis Studies (QUIPS) tool, and the domain-level risk-of-bias assessment is summarized in Figure 2. Overall, three studies were judged to have a low risk of bias, two had a moderate risk, and four had a high risk of bias. Outcome measurement was consistently rated as low risk across all studies, whereas confounding represented the principal source of bias, with three studies judged to have a high risk of bias. Most other domains were rated as low to moderate risk. Domain-level QUIPS assessments for individual studies are presented in Supplementary Table S3.

**Figure 3:**
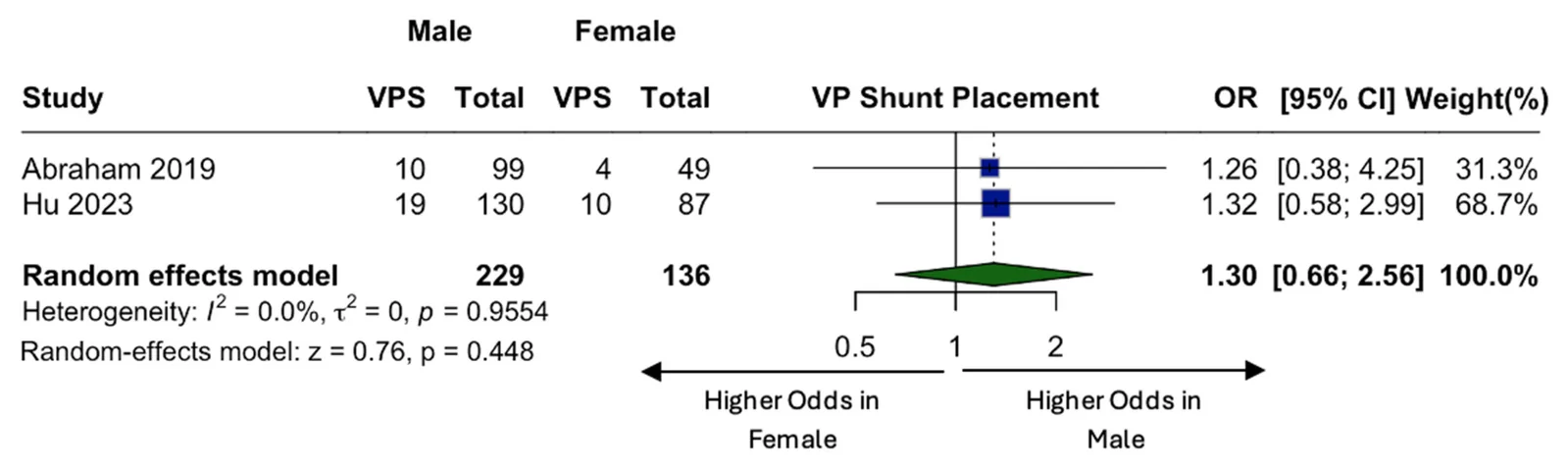
Sex as an associated factor of VP shunt placement following posterior fossa tumor resection in children [18,22].

Sex was evaluated in two studies comprising 365 children (229 males and 136 females). Male sex was not significantly associated with postoperative VP shunt placement compared with female sex (OR = 1.30, 95% CI 0.66–2.56; Figure 3). No between-study heterogeneity was observed (I^2^ = 0.0%, *p* = 0.96).

**Figure 4:**
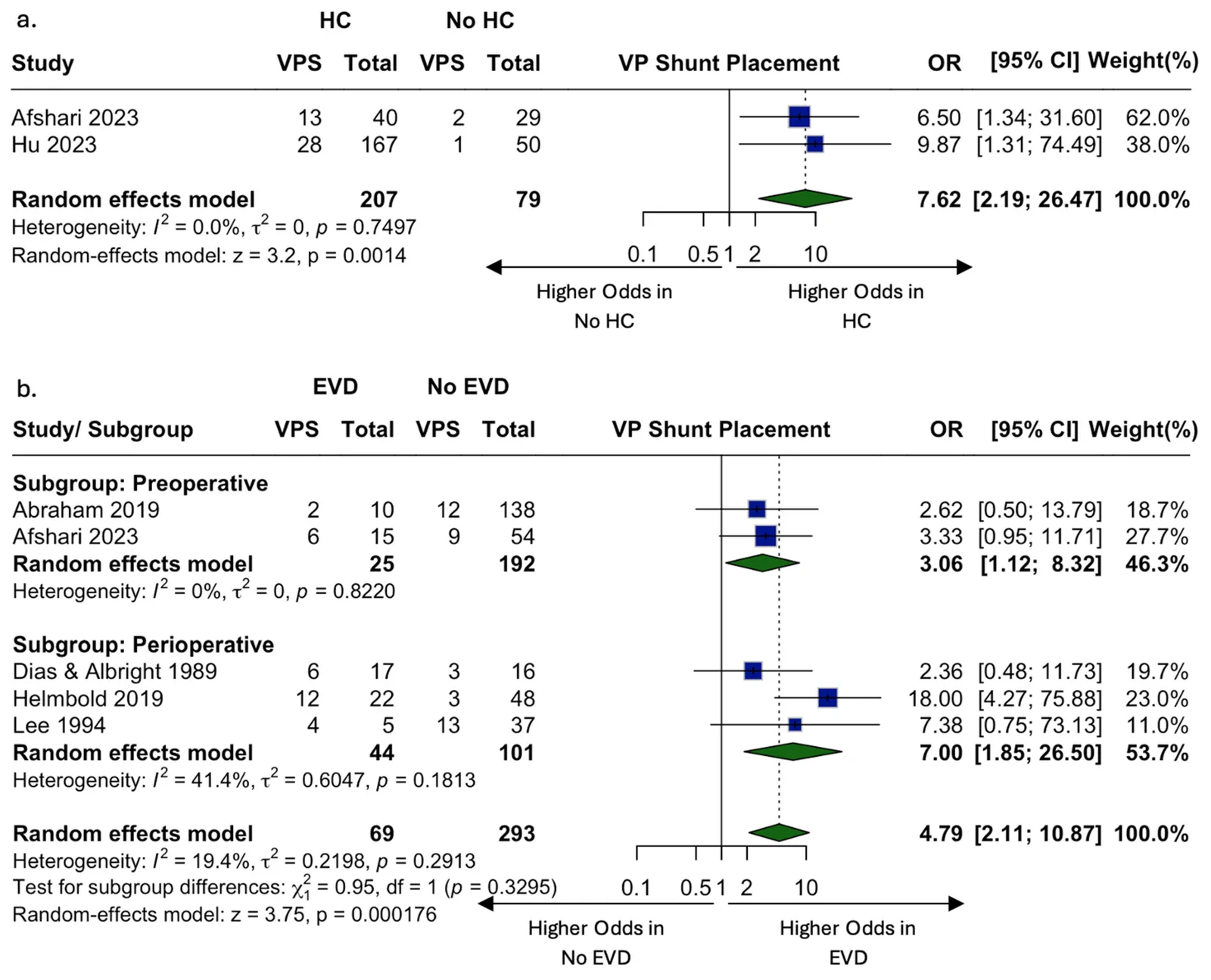
Hydrocephalus-related factors and management exposures associated with postoperative ventriculoperitoneal shunt placement following posterior fossa tumor resection in children. Forest plots showing the associations of (**a**) preoperative hydrocephalus and (**b**) external ventricular drain (EVD) exposure, including subgroup analyses of preoperative and perioperative EVD, with subsequent postoperative VP shunt placement. Squares represent study-specific ORs, with square size proportional to study weight; horizontal lines indicate 95% CIs, and diamonds represent pooled estimates [15,16,18,19,21,22].

Two studies comprising 286 children evaluated preoperative hydrocephalus. Children with preoperative hydrocephalus had significantly higher odds of postoperative VP shunt placement than those without hydrocephalus (OR = 7.62, 95% CI 2.19–26.47; Figure 4a), with no between-study heterogeneity (I^2^ = 0.0%, *p* = 0.75). Five studies comprising 362 children evaluated external ventricular drainage (EVD). EVD was associated with significantly higher odds of postoperative VP shunt placement compared with no EVD (OR = 4.79, 95% CI 2.11–10.87; Figure 4b), with low between-study heterogeneity (I^2^ = 19.4%, *p* = 0.29). In subgroup analyses, both preoperative EVD (2 studies; 217 children; OR = 3.06, 95% CI 1.12–8.32; I^2^ = 0.0%) and perioperative EVD (3 studies; 145 children; OR = 7.00, 95% CI 1.85–26.50; I^2^ = 41.4%) were significantly associated with higher odds of VP shunt placement. There was no significant difference between the subgroup estimates (*p* = 0.33).

**Figure 5:**
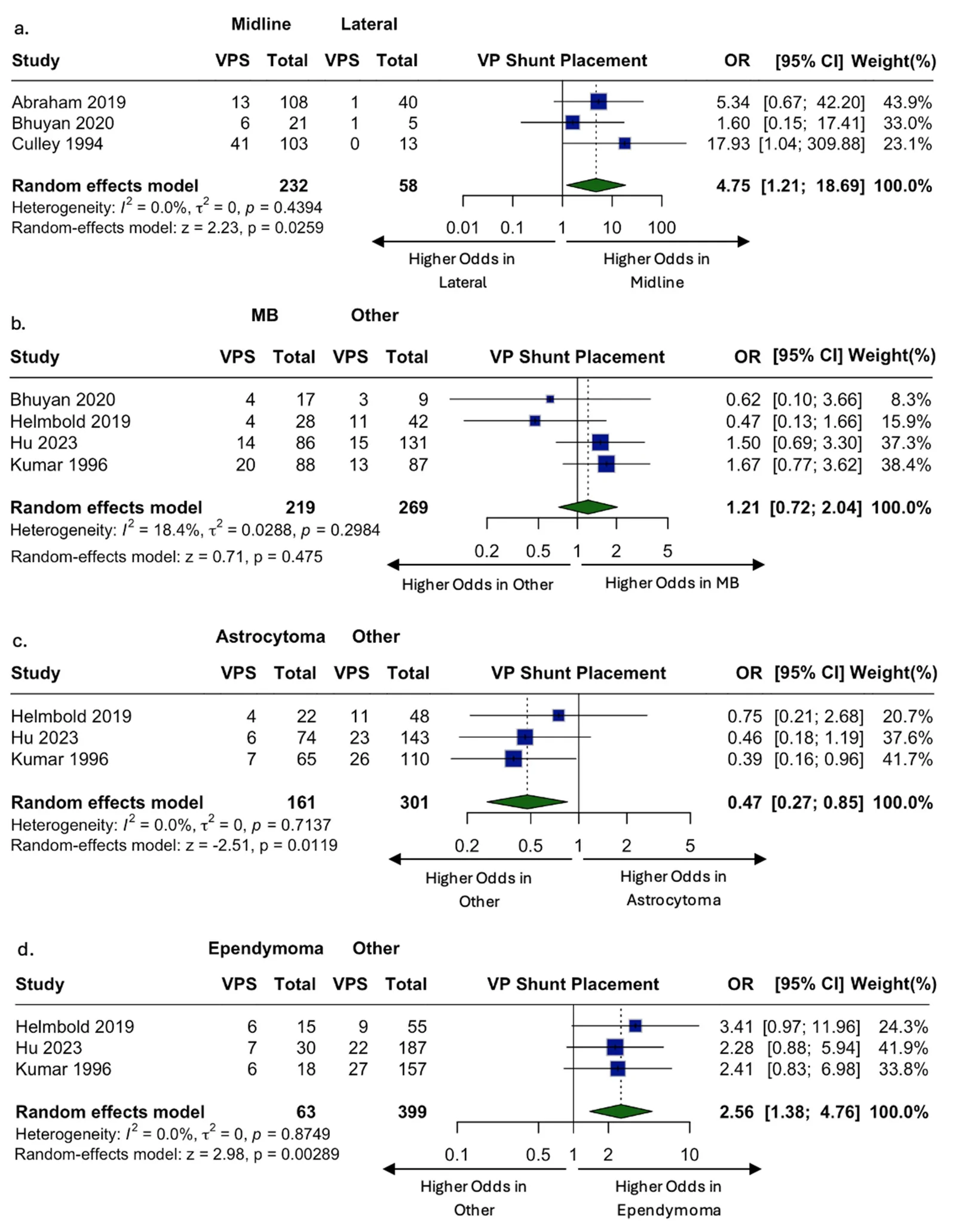
Tumor-related factors of ventriculoperitoneal shunt placement following posterior fossa tumor resection in children. Forest plots showing the associations between (**a**) tumor location, (**b**) medulloblastoma, (**c**) astrocytoma, and (**d**) ependymoma with postoperative ventriculoperitoneal shunt placement. Squares represent study-specific odds ratios (ORs), with square size proportional to study weight; horizontal lines indicate 95% confidence intervals (CIs), and diamonds represent pooled estimates. MB: Medulloblastoma [6,14,18,20,21,22].

Three studies involving 290 children compared midline with lateral posterior fossa tumors. Midline tumor location was associated with significantly higher odds of postoperative VP shunt placement compared with lateral tumor location (OR = 4.75, 95% CI 1.21–18.69; Figure 5a), with no between-study heterogeneity (I^2^ = 0.0%, *p* = 0.44). Four studies comprising 488 children evaluated medulloblastoma histology. Medulloblastoma was not significantly associated with postoperative VP shunt placement compared with other tumor histologies (OR = 1.21, 95% CI 0.72–2.04; Figure 5b), with low between-study heterogeneity (I^2^ = 18.4%, *p* = 0.30). Three studies comprising 462 children evaluated astrocytoma. Astrocytoma was associated with significantly lower odds of postoperative VP shunt placement compared with other tumor histologies (OR = 0.47, 95% CI 0.27–0.85; Figure 5c), with no between-study heterogeneity (I^2^ = 0.0%, *p* = 0.71). Three studies comprising 462 children evaluated ependymoma. Ependymoma was associated with significantly higher odds of postoperative VP shunt placement compared with other tumor histologies (OR = 2.56, 95% CI 1.38–4.76; Figure 5d), with no between-study heterogeneity (I^2^ = 0.0%, *p* = 0.87).

Among the nine included studies, additional tumor characteristics were reported in a limited number of studies and with heterogeneous definitions and categorizations, precluding quantitative synthesis; these factors were therefore synthesized narratively (Supplementary Table S4). Hu et al. [22] reported that WHO grade III–IV tumors were more frequent in the postoperative VP-shunt group than in the non-shunted group (75.9% vs. 50.5%; OR 3.08, *p* = 0.014), whereas tumor size >30 mm was not significantly associated with shunting (79.3% vs. 66.5%; OR 1.93, *p* = 0.173). In the same study, fourth-ventricular tumor location remained independently associated with postoperative VP shunt placement (aOR 7.70, 95% CI 2.60–22.84; *p* < 0.001). Abraham et al. found no independent association between their study-specific aggressive versus benign pathological classification and postoperative shunting [18]. Afshari et al. evaluated medulloblastoma molecular subgroup and found that molecular subgroup was not independently associated with postoperative VP shunt placement after adjustment [19].

**Figure 6:**
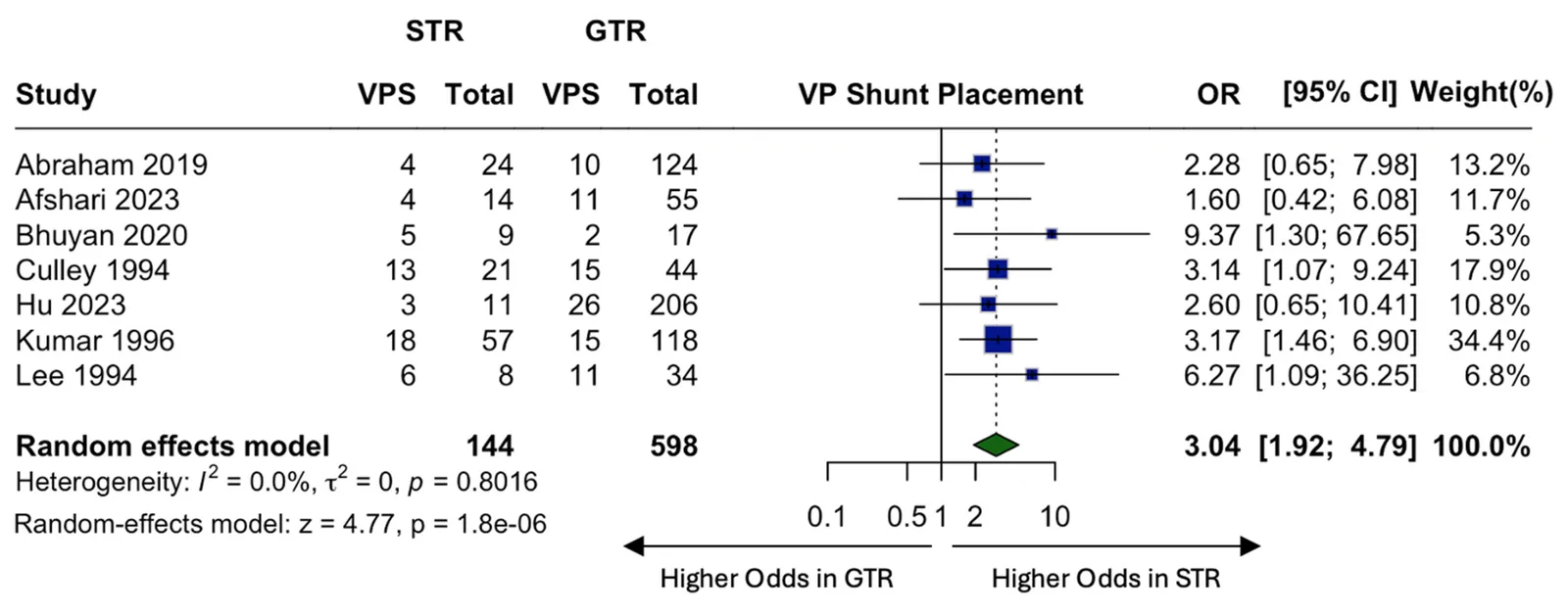
Extent of tumor resection as a factor of ventriculoperitoneal shunt placement following posterior fossa tumor resection in children. Forest plot showing the association between extent of tumor resection subtotal resection (STR) vs. gross total resection (GTR) and postoperative VP shunt placement. Squares represent study-specific ORs, with square size proportional to study weight; horizontal lines indicate 95% CIs, and diamonds represent pooled estimates [6,14,16,18,19,20,22].

Seven studies comprising 742 children compared subtotal resection (STR) with gross total resection (GTR). STR was associated with significantly higher odds of postoperative VP shunt placement compared with GTR (OR = 3.04, 95% CI 1.92–4.79; Figure 6). The pooled estimate demonstrated excellent consistency, with no evidence of between-study heterogeneity (I^2^ = 0.0%, *p* = 0.80).

**Figure 7:**
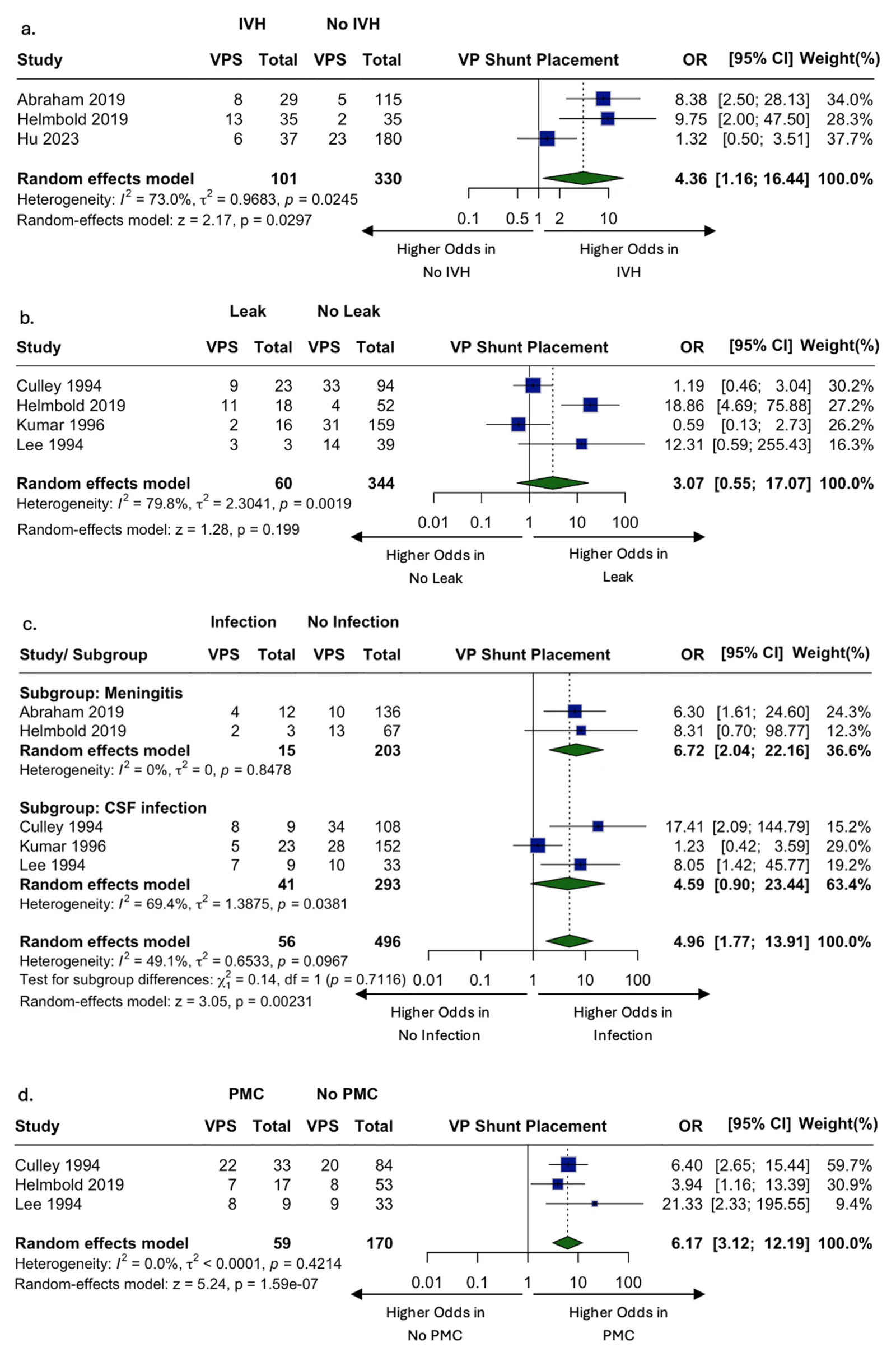
Postoperative factors of ventriculoperitoneal shunt placement following posterior fossa tumor resection in children. Forest plots showing the associations of (**a**) intraventricular hemorrhage, (**b**) cerebrospinal fluid (CSF) leakage, (**c**) postoperative infection, including subgroup analyses of meningitis and CSF infection, and (**d**) pseudomeningocele with postoperative ventriculoperitoneal shunt placement. Squares represent study-specific ORs, with square size proportional to study weight; horizontal lines indicate 95% CIs, and diamonds represent pooled estimates [6,14,16,18,21,22].

Three studies (*n* = 431 children) evaluated postoperative intraventricular hemorrhage (IVH). Across these three studies, postoperative IVH was associated with significantly higher odds of VP shunt placement compared with the absence of IVH (OR = 4.36, 95% CI 1.16–16.44; Figure 7a), although the estimate was imprecise and should be interpreted cautiously given the limited number of contributing studies. Substantial between-study heterogeneity was observed (I^2^ = 73.0%, *p* = 0.02). Four studies comprising 404 children evaluated postoperative cerebrospinal fluid (CSF) leakage. CSF leakage was not significantly associated with postoperative VP shunt placement compared with no CSF leakage (OR = 3.07, 95% CI 0.55–17.07; Figure 7b). Considerable between-study heterogeneity was observed (I^2^ = 79.8%, *p* = 0.002). Postoperative infection was evaluated across six study comparisons comprising 552 children. Overall, postoperative infection was associated with significantly higher odds of VP shunt placement compared with no postoperative infection (OR = 4.96, 95% CI 1.77–13.91; Figure 7c), with moderate between-study heterogeneity (I^2^ = 49.1%, *p* = 0.10). In subgroup analyses, postoperative meningitis was associated with significantly higher odds of VP shunt placement (2 studies; 218 children; OR = 6.72, 95% CI 2.04–22.16; I^2^ = 0.0%). CSF infection showed a similar direction of association but did not reach statistical significance (3 studies; 334 children; OR = 4.59, 95% CI 0.90–23.44; I^2^ = 69.4%). There was no significant difference between the meningitis and CSF infection subgroup estimates (*p* = 0.71). Three studies involving 229 children evaluated postoperative pseudomeningocele. Across these three studies, pseudomeningocele was associated with significantly higher odds of postoperative VP shunt placement compared with no pseudomeningocele (OR = 6.17, 95% CI 3.12–12.19; Figure 7d). No between-study heterogeneity was observed (I^2^ = 0.0%, *p* = 0.42); however, the association was based on only three studies and should therefore be interpreted cautiously.

Several factor-specific estimates were supported by a limited number of studies, with most derived from only two or three contributing studies, warranting cautious interpretation of the pooled estimates.

Sensitivity analyses were performed to assess the robustness of the pooled findings and the potential influence of study era. Leave-one-out analyses demonstrated that the association between pseudomeningocele and VP shunt placement remained robust, with pooled ORs ranging from 5.42 to 7.54 and all 95% CIs excluding unity (Figure S1). The association for ependymoma was similarly robust, with pooled ORs ranging from 2.34 to 2.78 (Figure S2). In contrast, the association for midline tumor location was sensitive to individual-study exclusion, remaining statistically significant only when Bhuyan et al. was omitted (Figure S3). Postoperative CSF leakage remained non-significant and imprecise across all leave-one-out analyses (Figure S4), while medulloblastoma remained consistently non-significant (Figure S5). The inverse association observed for astrocytoma was less robust, remaining statistically significant after exclusion of Helmbold et al. but not after exclusion of Hu et al. or Kumar et al. (Figure S6). Temporal subgroup analyses showed no significant difference between contemporary and older studies for EVD exposure (OR = 5.47, 95% CI 1.69–17.70 vs. OR = 3.43, 95% CI 0.92–12.77; *p* for subgroup difference = 0.60; Figure S7) or subtotal resection (OR = 2.56, 95% CI 1.26–5.22 vs. OR = 3.42, 95% CI 1.89–6.19; *p* for subgroup difference = 0.54; Figure S8). Factor-specific sensitivity analyses excluding contributing historical cohorts published between 1989 and 1996 showed that several primary findings remained robust. EVD exposure remained associated with higher odds of subsequent VP shunt placement (OR 5.47, 95% CI 1.69–17.70; I^2^ = 49.8%), as did ependymoma (OR 2.64, 95% CI 1.24–5.66; I^2^ = 0%), incomplete tumor resection (OR 2.56, 95% CI 1.26–5.22; I^2^ = 0%), and postoperative infection (OR 6.72, 95% CI 2.04–22.16; I^2^ = 0%). Medulloblastoma remained non-significant (OR 0.90, 95% CI 0.39–2.10; I^2^ = 25.8%). In contrast, the associations for midline tumor location (OR 3.18, 95% CI 0.67–15.20; I^2^ = 0%) and astrocytoma (OR 0.55, 95% CI 0.26–1.17; I^2^ = 0%) were attenuated and no longer statistically significant. Overall, most associations remained directionally consistent, although the findings for midline tumor location and astrocytoma were sensitive to the inclusion of historical cohorts (Figures S9–S16).

The certainty of evidence for factors and exposures associated with postoperative VP shunt placement ranged from very low to low (Table 2). Limited availability of comparable adjusted estimates and concerns regarding residual confounding contributed to the study-limitations judgments and factor-specific certainty ratings. Low-certainty evidence was found for male sex, preoperative hydrocephalus, EVD exposure, midline tumor location, medulloblastoma, astrocytoma, ependymoma, subtotal resection, postoperative infection, and pseudomeningocele. Evidence for postoperative IVH was rated as having very low certainty because of study limitations, substantial between-study heterogeneity, and imprecision. Evidence for postoperative CSF leakage was also rated very low certainty because of study limitations, substantial heterogeneity, and marked imprecision, with the confidence interval encompassing both no association and a potentially large increase in the odds of VP shunt placement. Although several pooled associations were of moderate or large magnitude, effect size was not used to upgrade certainty because all pooled estimates were derived from unadjusted data and residual confounding could not be excluded. Publication bias could not be reliably assessed because fewer than 10 studies contributed to each factor-specific meta-analysis.

## 4. Discussion

This systematic review and meta-analysis synthesized evidence from nine retrospective cohort studies involving 922 children undergoing posterior fossa tumor resection. Preoperative hydrocephalus, external ventricular drainage (EVD), midline tumor location, ependymoma histology, subtotal tumor resection, postoperative intraventricular hemorrhage (IVH), postoperative infection, and pseudomeningocele were significantly associated with higher odds of postoperative ventriculoperitoneal shunt (VPS) placement. In contrast, astrocytoma was associated with lower odds of VP shunt placement, whereas sex, medulloblastoma histology, and postoperative cerebrospinal fluid (CSF) leakage were not significantly associated with the outcome. Importantly, all 12 primary factor-specific meta-analyses were derived from crude/unadjusted event data. Although several individual studies reported multivariable or partially adjusted analyses, sufficiently comparable adjusted estimates were unavailable for quantitative pooling. The pooled ORs should therefore be interpreted as crude associations with subsequent VP shunt placement rather than independent prognostic or causal effects, particularly because several evaluated factors are clinically interrelated and residual confounding is likely.

The certainty-of-evidence assessment provides important context for interpreting these associations. Overall, certainty ranged from very low to low, reflecting the retrospective nature of the included studies, the limited number of studies contributing to several analyses, inconsistent adjustment for potential confounders, and reliance of the pooled analyses on crude event data. Although several associations showed moderate or large pooled ORs, effect magnitude was not considered sufficient to upgrade certainty because residual confounding could not be excluded. Low-certainty evidence was identified for most evaluated factors and exposures, including pseudomeningocele, whereas certainty was very low for postoperative IVH and CSF leakage because of study limitations, substantial between-study heterogeneity, and imprecision. Accordingly, the pooled estimates should be interpreted as associations rather than independent prognostic effects, particularly where confidence intervals were wide or residual confounding was likely. Differences in the radiological and clinical definitions of hydrocephalus across the included studies further limit the comparability and interpretation of these associations.

Preoperative hydrocephalus demonstrated the strongest pooled association among the preoperative factors evaluated. Children with hydrocephalus before tumor resection had more than sevenfold higher odds of postoperative VP shunt placement than those without preoperative hydrocephalus. This association is biologically plausible because pronounced or prolonged ventricular enlargement may reflect severe CSF pathway obstruction, reduced intracranial compliance, and impaired CSF absorption that may not immediately normalize after tumor removal. Quantitative radiological studies have similarly shown that greater preoperative ventricular enlargement, including elevated Evans Index and fronto-occipital horn ratio values, is associated with persistent postoperative hydrocephalus [23]. Severe preoperative hydrocephalus was also associated with persistent hydrocephalus in a large pediatric posterior fossa tumor cohort managed with early tumor surgery [24]. More recently, preoperative hydrocephalus was retained as an independent component of a validated clinical scoring system for postoperative VP shunt requirement, further supporting its prognostic relevance [25].

Younger age also emerged as a potentially important factor associated with subsequent VP shunt placement, although quantitative synthesis was not feasible because of heterogeneity in age definitions, thresholds, and reporting across studies. Abraham et al. identified age ≤6 years as independently associated with subsequent shunting (adjusted OR 5.9, 95% CI 1.6–22.6), while Hu et al. reported an independent association for age <3 years (adjusted OR 3.76, 95% CI 1.29–10.93) [18,22]. Other studies also evaluated younger age using varying thresholds, limiting direct comparison and precluding a reliable pooled estimate. These findings, together with the previous prognostic literature identifying younger age as relevant to postresection hydrocephalus, suggest that age remains a potentially important factor despite its exclusion from the quantitative synthesis. Future studies using standardized age definitions and appropriately adjusted analyses are needed to clarify its independent association with subsequent VP shunt placement.

**Figure 8:**
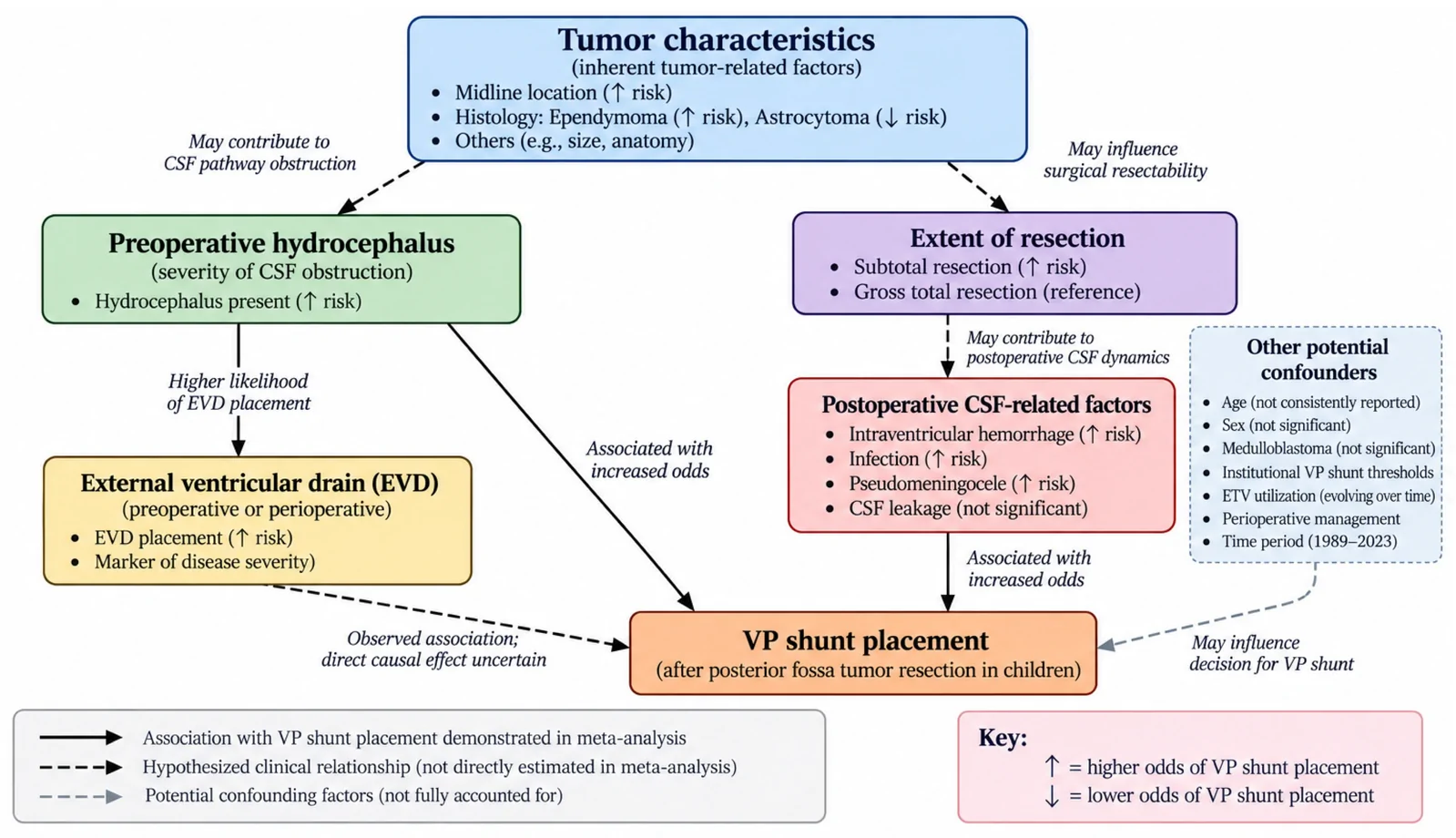
Conceptual framework illustrating factors associated with VP shunt placement and potential confounding by indication in the EVD association. The framework summarizes factors evaluated in the meta-analysis and illustrates potential relationships among tumor characteristics, hydrocephalus severity, EVD placement, and subsequent VP shunt placement. Solid arrows indicate associations with VP shunt placement demonstrated in the meta-analysis, whereas dashed arrows represent hypothesized clinical pathways that were not directly estimated. The relationship between EVD exposure and subsequent VP shunt placement should be interpreted cautiously because hydrocephalus severity may influence both EVD placement and subsequent shunt requirement, resulting in confounding by indication. The framework is intended to aid interpretation and does not establish causal relationships.

EVD use was also associated with significantly higher odds of postoperative VP shunt placement, with significant associations observed for both preoperative and perioperative EVD. However, there was no significant difference between these subgroup estimates, suggesting no evidence that the magnitude of the association differed according to EVD timing. This finding should be interpreted cautiously because EVD placement is unlikely to represent a direct cause of subsequent VP shunt requirement. Rather, EVD use may reflect more severe obstructive hydrocephalus, uncontrolled intracranial hypertension, or greater perioperative neurological compromise. Prolonged EVD requirement has previously been associated with subsequent shunt placement, supporting its role as a marker of persistent CSF dysregulation rather than an isolated causal factor [14]. Perioperative EVD was likewise associated with VP shunt placement in a contemporary pediatric cohort [21]. A pilot comparison of EVD and no-EVD strategies reported a higher frequency of subsequent permanent CSF diversion among EVD-treated patients, although the non-randomized design and treatment-selection bias precluded causal inference [26]. The potential relationships among tumor characteristics, hydrocephalus severity, EVD placement, and subsequent VP shunt placement, including the potential for confounding by indication, are illustrated in the conceptual framework presented in Figure 8.

The narrative synthesis provides additional insight into tumor-related characteristics that were not suitable for quantitative pooling. Within the included studies, fourth-ventricular tumor location emerged as the most notable anatomical factor, retaining an independent association with postoperative VP shunt placement in Hu et al. [22]. Conversely, the association observed for WHO grade III–IV in the same cohort was not retained in the multivariable model, while tumor size >30 mm, aggressive versus benign pathological classification in Abraham et al. [18], and medulloblastoma molecular subgroup in Afshari et al. did not show independent associations [19]. Collectively, these findings suggest a potential role for tumor anatomy, particularly involvement of the fourth ventricle and its relationship to CSF pathways, in subsequent shunt requirement. Nevertheless, the small number of contributing studies, heterogeneity in variable definitions and categorizations, and differences in adjustment strategies precluded quantitative pooling and limited firm conclusions regarding these characteristics as independent prognostic factors.

Midline tumor location was associated with almost fivefold higher odds of postoperative VP shunt placement compared with lateral tumor location. This relationship is anatomically plausible because midline lesions commonly involve the cerebellar vermis, fourth ventricle, and ventricular outlets, resulting in direct and potentially prolonged obstruction of CSF circulation. Earlier studies similarly identified midline tumor location as a factor associated with postoperative shunting [14]. Large institutional data also demonstrate that vermian and fourth ventricular tumors have particularly high rates of obstructive hydrocephalus at presentation [27]. More recent multivariable prediction research has identified midline location as an independent factor associated with postoperative VP shunt placement [25]. Nevertheless, the pooled analysis was based on only three studies, and the association should therefore be considered suggestive rather than definitive.

Histological subtype was also associated with VP shunt placement. Ependymoma was associated with higher odds of VP shunt placement, whereas astrocytoma was associated with lower odds. Ependymomas commonly arise from the fourth ventricular region and may extend along the ventricular floor and outlets, potentially producing persistent distortion or obstruction of CSF pathways. In contrast, the astrocytoma populations represented in pediatric posterior fossa series frequently include pilocytic astrocytomas, which commonly arise within the cerebellum and may have less direct involvement of the fourth ventricular CSF pathways. Their generally circumscribed growth pattern may also permit more complete relief of mechanical CSF obstruction following resection. Kumar et al. reported that astrocytomas were more frequently laterally located and were associated with a lower requirement for postoperative shunting [6]. Hydrocephalus has also been reported to resolve more frequently following astrocytoma resection than after medulloblastoma or ependymoma resection [28]. These anatomical and histological differences may partly explain the observed inverse association; however, astrocytoma should not be interpreted as independently protective because histological subtype, tumor location, CSF pathway involvement, and extent of resection are interrelated and were not consistently adjusted for across studies.

Medulloblastoma was not significantly associated with postoperative VP shunt placement compared with other tumor histologies. This finding differs from several earlier reports in which medulloblastoma was considered a high-risk histology. In a medulloblastoma-specific cohort, younger age, greater ventricular enlargement, and more advanced tumor extent were associated with permanent shunting, suggesting that disease severity and anatomical burden may be more influential than histological diagnosis alone [16]. The absence of a significant pooled association in the present analysis suggests that medulloblastoma histology alone may have limited prognostic value without consideration of concurrent factors such as ventricular obstruction, metastatic dissemination, and preoperative hydrocephalus.

Subtotal resection was associated with approximately threefold higher odds of postoperative VP shunt placement compared with gross total resection, with no detectable between-study heterogeneity. Earlier studies similarly reported a greater requirement for CSF diversion following incomplete tumor removal [6,14,15]. Residual tumor may continue to compromise CSF pathways; however, subtotal resection may also serve as a marker of tumors that are larger, more infiltrative, or closely related to critical ventricular structures. Partial resection was likewise identified as an associated factor of persistent hydrocephalus in children with periventricular tumors (32*). Importantly, this association should not be interpreted as justification for greater surgical aggressiveness, because the extent of resection must remain balanced against neurological preservation and oncological objectives. Therefore, the association may partly reflect confounding by tumor anatomy and biological complexity, rather than an independent effect of the extent of resection itself.

The associations of postoperative IVH, infection, and pseudomeningocele with VP shunt placement should be interpreted cautiously because these events occur during the evolving postoperative course. Rather than representing independent prognostic factors, they may contribute to impaired CSF circulation, reflect persistent hydrocephalus, or arise as part of the same clinical trajectory leading to permanent CSF diversion. Previous pediatric studies have reported associations of postoperative intraventricular blood, CSF infection, and pseudomeningocele with subsequent hydrocephalus or shunt requirement [14,18]. Importantly, CSF leakage and tense pseudomeningocele may themselves be manifestations of persistent hydrocephalus and have been reported as clinical indications for shunt placement [18]. Because the included studies did not consistently establish the timing of these events relative to hydrocephalus progression and VP shunt placement, independent prognostic or causal effects cannot be inferred.

Postoperative IVH was associated with significantly higher odds of VP shunt placement; however, substantial between-study heterogeneity was observed, warranting cautious interpretation of the magnitude of this association. Intraventricular blood may obstruct ventricular outlets and induce inflammatory changes that impair CSF absorption. Postoperative intraventricular blood was associated with shunt placement in the cohort reported by Helmbold et al. (2019) [21]. Hu et al. also identified intraoperative blood loss as an associated factor of postoperative VP shunt placement, supporting a potential contribution of perioperative hemorrhagic burden to persistent hydrocephalus [22]. A recent study of tumors involving or adjacent to CSF pathways similarly found postoperative intraventricular hematoma to be independently associated with VP shunt requirement [29]. The substantial heterogeneity observed in the present analysis may reflect differences in hemorrhage definition, volume, timing, and imaging assessment across studies.

Postoperative infection was associated with higher odds of VP shunt placement in the overall analysis; however, meningitis and CSF infection were analyzed separately as clinically distinct subgroups. Meningitis was significantly associated with VP shunt placement, whereas the CSF-infection subgroup showed a similar direction of effect but did not reach statistical significance and demonstrated substantial heterogeneity. Importantly, there was no significant difference between the subgroup estimates. Infection may impair CSF absorption through meningeal inflammation, arachnoid fibrosis, and obstruction by inflammatory debris. CSF infection was previously associated with postoperative shunt placement in one large pediatric cohort [14], whereas another series did not demonstrate a statistically significant association [6]. Differences in diagnostic criteria, microbiological confirmation, timing of infection, and antimicrobial treatment may contribute to the variability across studies.

Pseudomeningocele was strongly associated with postoperative VP shunt placement, with no detectable between-study heterogeneity. A pseudomeningocele may represent an external manifestation of persistently abnormal CSF pressure or impaired CSF circulation rather than an isolated wound complication. Persistent pseudomeningocele was associated with subsequent shunt placement in the cohort of Culley et al. (1994) [14]. Kumar et al. also reported persistent hydrocephalus, CSF leakage, and pseudomeningocele among the clinical indications for postoperative shunting [6]. The consistency of the pooled association suggests that postoperative pseudomeningocele may represent an important clinical warning sign for persistent CSF dysregulation. This finding was also robust in leave-one-out sensitivity analysis, with the association remaining significant after sequential exclusion of each contributing study.

Postoperative CSF leakage was not significantly associated with VP shunt placement, although the point estimate was in the direction of higher VP shunt odds among patients with CSF leakage. The wide confidence interval and considerable between-study heterogeneity indicate substantial uncertainty regarding this association. CSF leakage may result from dural closure technique, impaired wound healing, elevated intracranial pressure, or localized postoperative CSF accumulation and therefore represents a clinically heterogeneous complication. Muszynski et al. found no significant effect of perioperative ventricular drainage or dural closure technique on postoperative CSF leakage [30]. Recent data also indicate that CSF leakage, pseudomeningocele, and postoperative hydrocephalus should not necessarily be considered equivalent manifestations of the same pathological process [31]. Leave-one-out sensitivity analysis did not materially resolve this uncertainty; the association remained non-significant and imprecise after sequential omission of individual studies, indicating that the finding is not sufficiently robust to support a consistent association with VP shunt placement.

Male sex was not significantly associated with postoperative VP shunt placement, and no between-study heterogeneity was observed. Although the pooled point estimate was in the direction of higher VP shunt odds among males, the confidence interval crossed the null. These findings provide no evidence of a sex-based association with VP shunt placement; however, only two studies contributed to this analysis, limiting the precision and certainty of the estimate.

### Clinical Implications

The present findings may support a risk-informed approach to postoperative surveillance rather than prophylactic permanent CSF diversion. Children with preoperative hydrocephalus, midline tumors, ependymoma, or subtotal resection may warrant closer postoperative neurological and imaging surveillance and earlier reassessment for persistent hydrocephalus when clinically indicated. In patients requiring temporary CSF diversion, careful assessment of ventricular dynamics and clinical status during EVD weaning may be particularly important. Postoperative IVH, infection, or pseudomeningocele may further identify patients requiring closer evaluation for persistent CSF dysregulation. However, the generally low certainty of evidence precludes the use of any individual factor as an indication for VP shunt placement.

Importantly, the observed associations should not be interpreted as causal or used individually as indications for VP shunt placement. Most pooled estimates were derived from unadjusted data, and several factors may reflect underlying disease severity or treatment selection rather than independent biological effects. EVD use, for example, may primarily reflect the severity of hydrocephalus and treatment selection rather than an independent biological effect, whereas subtotal resection may reflect complex tumor anatomy. Decisions regarding definitive CSF diversion should therefore integrate clinical symptoms, ventricular trajectory, wound findings, response to temporary CSF drainage, and the timing and requirements of oncological treatment.

Both VP shunt and endoscopic third ventriculostomy may be appropriate for selected children with established persistent hydrocephalus. A multicenter Hydrocephalus Clinical Research Network study found similar overall treatment-failure rates for VP shunt and endoscopic third ventriculostomy, although endoscopic third ventriculostomy failures occurred earlier, whereas VP shunt failures continued over a longer follow-up period [1]. Procedure selection should therefore consider the mechanism of hydrocephalus, tumor characteristics, expected clinical course, institutional expertise, and the distinct failure profiles of each intervention.

An additional consideration is that VP shunt placement represents a clinical management decision rather than a purely biological endpoint. Thresholds for permanent CSF diversion may vary according to surgeon judgment, institutional practice, EVD-weaning protocols, symptom severity, ventricular progression, and alternative CSF-diversion strategies such as ETV. Consequently, VP shunt placement may not represent a uniform surrogate for persistent postoperative hydrocephalus across all included studies. This variability should be considered when interpreting differences in VP shunt incidence and associated factors between cohorts.

Factor-specific sensitivity analyses, excluding contributing historical cohorts published between 1989 and 1996 [6,14,15,16], provided additional insight into the influence of the study era. Associations for EVD exposure, ependymoma, incomplete tumor resection, and postoperative infection remained statistically significant, whereas medulloblastoma remained non-significant. In contrast, the associations for midline tumor location and astrocytoma were attenuated and no longer statistically significant, suggesting greater sensitivity to historical data. These findings are relevant given the evolution of neuroimaging, surgical techniques, perioperative care, CSF-diversion practices, and thresholds for permanent VP shunt placement over time. Accordingly, estimates that were not robust to historical-cohort exclusion should be interpreted cautiously.

Several limitations should be acknowledged. All included studies were retrospective, increasing susceptibility to selection bias and residual confounding. A major limitation is that all pooled factor-specific estimates were derived from crude event data rather than consistently adjusted effect estimates. Although four studies reported formal multivariable analyses and two used partial or stratified adjustment, substantial heterogeneity in the factors evaluated, covariate sets, statistical methods, and reported effect measures precluded meaningful pooling of adjusted estimates. Consequently, residual confounding remains an important concern, particularly because tumor characteristics, hydrocephalus severity, EVD placement, extent of resection, and postoperative CSF-related factors are clinically interrelated. This is especially relevant to EVD, which represents a therapeutic intervention and may serve as a marker of underlying hydrocephalus severity and treatment selection, making the observed association with subsequent VP shunt placement susceptible to confounding by indication. Accordingly, the pooled ORs should be interpreted as crude associations rather than independent prognostic or causal effects. Clinical and methodological heterogeneity across studies should also be considered when interpreting the magnitude and generalizability of these associations. The included studies spanned more than three decades, during which neuroimaging, surgical techniques, EVD and ETV practices, and neuro-oncological management evolved. Definitions of hydrocephalus and institutional or surgeon thresholds for VP shunt placement also varied. Consequently, VP shunt placement is a management-dependent outcome and may not uniformly represent persistent hydrocephalus across studies. Several pooled analyses were based on only two or three studies, and substantial heterogeneity was observed for postoperative IVH and CSF leakage, limiting the robustness of these estimates. Age and other potentially relevant variables, including quantitative ventricular indices, tumor volume, molecular subtype, metastatic dissemination, intraoperative blood loss, and postoperative ventricular trajectory, could not be pooled because of insufficient or inconsistent reporting. Formal assessment of publication bias and small-study effects was not reliable because fewer than 10 studies contributed to each factor-specific meta-analysis [17]; however, small-study effects, selective reporting of significant associations, and underpublication of negative findings cannot be excluded. Collectively, these limitations were reflected in the factor-specific GRADE assessments, with the certainty of evidence ranging from very low to low.

Prospective multicenter studies should apply standardized definitions of preoperative hydrocephalus, persistent postoperative hydrocephalus, EVD exposure, infection, CSF leakage, pseudomeningocele, and indications and timing of VP shunt placement. Future prognostic studies should prioritize multivariable analyses reporting adjusted effect estimates and explicitly address confounding by disease severity, tumor characteristics, and treatment selection. Standardized reporting of the timing of VP shunt placement would also facilitate evaluation of early versus delayed shunt requirement. Externally validated prediction models integrating clinical characteristics, quantitative ventricular measurements, tumor anatomy and molecular features, extent of resection, and postoperative complications may improve individualized estimation of VP shunt requirement. Such models should support postoperative surveillance and multidisciplinary decision-making rather than serve as automatic indications for prophylactic VP shunt placement.

## 5. Conclusions

Several clinical, tumor-related, operative, and postoperative factors were associated with subsequent VP shunt placement following posterior fossa tumor resection in children. Preoperative hydrocephalus, EVD exposure, midline tumor location, ependymoma, subtotal resection, postoperative IVH, infection, and pseudomeningocele were associated with higher odds of VP shunt placement, whereas astrocytoma was associated with lower odds. Sex, medulloblastoma, and postoperative CSF leakage were not significantly associated. Importantly, VP shunt placement is a management-dependent outcome and should not be regarded as a direct surrogate for persistent hydrocephalus. The observed associations may reflect both underlying CSF dysfunction and variation in institutional practices, CSF-diversion strategies, and thresholds for permanent shunting. Given the predominantly low-certainty, unadjusted evidence, these findings represent associations with VP shunt placement rather than independent prognostic or causal determinants of persistent hydrocephalus. Future prospective multicenter studies with standardized definitions, CSF-diversion protocols, and robust multivariable adjustment are needed to determine whether these associations represent independent prognostic factors and to support the development of reliable risk-stratification models.

## Figures and Tables

**Table 1:** Characteristics of included studies and clinically relevant baseline or outcomes characteristics.

Study	Country/Center	Study Period	Population/N	Age	Preoperative Hydrocephalus	Postoperative VPS Placement	Follow-Up/Timing of VPS	Main Tumor Population
Abraham et al., 2019 [18]	India, single center	Jan 2005–Sep 2018	Children < 18 y undergoing primary PFT resection; N = 148	Median 8 y (IQR 5–12); mean 8.7 y	141/148 (95.3%) radiographic HC; 131/141 symptomatic	14/148 (9.4%)	Minimum follow-up ≥30 d; median VPS timing 14 d (IQR 6.7–17.2)	Mixed PFT; medulloblastoma 51.3%, pilocytic astrocytoma 35.8%, ependymoma 8.8%
Afshari et al., 2023 [19]	UK/Hong Kong, 3 tertiary centers	4-year study period; exact calendar dates NR	Children 0–17 y with medulloblastoma and molecular data; N = 69	Mean 6.2 y	40/69 (58.0%) derived from 13/15 shunted + 27/54 non-shunted	15/69 (21.7%)	Overall follow-up duration NR; median time to VPS 0.5 mo (range 0.13–27.6)	Medulloblastoma only; WNT, SHH, Group 3, Group 4, Group 3/4
Bhuyan et al., 2020 [20]	India, single center	Nov 2017–Aug 2019	Children < 14 y after PFT resection; N = 26	Mean 8.58 ± 4.62 y	Frequency NR; severity quantified using ventricular measurements	7/26 (26.9%)	NR	Medulloblastoma 65.4%, ependymoma 23.1%, astrocytoma 11.5%
Culley et al., 1994 [14]	USA, single center	1976–1990	Pediatric PFT cohort; N = 117	4 mo–16 y 9 mo	97/117 (82.9%) documented radiographically; severity documented in subset	42/117 (35.9%)	69% of VPS placed within 30 d; overall follow-up duration NR	Mixed PFT
Dias & Albright, 1989 [15]	USA, single center	1979–1987	Children with PFT-associated hydrocephalus; N = 58 evaluable	Pediatric cohort	100% had tumor-associated hydrocephalus by study selection	Of 33 initially non-shunted patients, 9/33 (27.3%) subsequently required permanent shunting; 25 had VPS before/at initial management	Mean follow-up approximately 52 mo (range 4 mo–8 y)	Mixed PFT
Helmbold et al., 2019 [21]	Germany, single center	Jan 2009–Jan 2018	Children/young patients with PFT; N = 70	Median 8.2 y; range 0.4–20.8	51/70 (72.9%)	15/70 (21.4%)	Mean VPS timing 16.6 d, median 16 d (range 7–27)	Mixed PFT
Hu et al., 2023 [22]	China, single center	Nov 2010–Dec 2020	Children ≤ 14 y after PFT resection; N = 217	Pediatric ≤ 14 y	167/217 (77.0%)	29/217 (13.4%)	21/29 within 2 weeks; additional cases occurred later, including at 6 and 12 mo; overall follow-up NR	Mixed PFT; medulloblastoma 39.6%, astrocytoma 34.1%, ependymoma 13.8%
Kumar et al., 1996 [6]	UK, single center	Jan 1983–Dec 1993	Children with PFT; N = 175	4 mo–14 y 6 mo	165/175 (94.3%)	33/175 (18.9%)	Minimum follow-up 1 y; majority of VPS inserted within 45 d	Medulloblastoma 50%, astrocytoma 37%, ependymoma 10%
Lee et al., 1994 [16]	USA, single center	1979–1990	Children with medulloblastoma without precraniotomy shunting; N = 42	Mean 8.1 ± 5.3 y	Preoperative hydrocephalus severity quantitatively assessed; exact overall HC frequency NR	17/42 (40.5%) within 4 weeks; 2 additional late shunts occurred at recurrence	Primary analysis: VPS within 4 weeks; longer overall follow-up duration NR	Medulloblastoma only

Note: HC, hydrocephalus; IQR, interquartile range; NR, not reported; PFT, posterior fossa tumor; VP Shunt, ventriculoperitoneal shunt, y, year, mo, month. For Dias and Albright, postoperative VP shunt incidence refers to the initially non-shunted subgroup because 25 patients underwent permanent VP shunt as part of initial hydrocephalus management. Helmbold et al. included a predominantly pediatric cohort (median age 8.2 years), although the reported age range extended to 20.8 years.

**Table 2:** GRADE assessment of the certainty of evidence for factors and exposures associated with VP shunt placement following PFT resection in children.

Factor/Exposure	Studies/ Participants	Pooled OR (95% CI)	Study Limitations	Inconsistency	Indirectness	Imprecision	Publication Bias	Magnitude of Association *	Certainty of Evidence
Male sex	2/365	1.30 (0.66–2.56)	Serious	Not serious	Not serious	Serious	Not assessed †	None	Low ⨁⨁◯◯
Preoperative hydrocephalus	2/286	7.62 (2.19–26.47)	Serious	Not serious	Not serious	Serious	Not assessed †	Large	Low ⨁⨁◯◯
EVD exposure	5/362	4.79 (2.11–10.87)	Serious ‡	Not serious	Not serious	Serious	Not assessed †	Large	Low ⨁⨁◯◯
Midline tumor location	3/290	4.75 (1.21–18.69)	Serious	Not serious	Not serious	Serious	Not assessed †	Large	Low ⨁⨁◯◯
Medulloblastoma	4/488	1.21 (0.72–2.04)	Serious	Not serious	Not serious	Serious	Not assessed †	None	Low ⨁⨁◯◯
Astrocytoma	3/462	0.47 (0.27–0.85)	Serious	Not serious	Not serious	Not serious	Not assessed †	Moderate §	Low ⨁⨁◯◯
Ependymoma	3/462	2.56 (1.38–4.76)	Serious	Not serious	Not serious	Not serious	Not assessed †	Moderate	Low ⨁⨁◯◯
Subtotal resection	7/742	3.04 (1.92–4.79)	Serious	Not serious	Not serious	Not serious	Not assessed †	Moderate	Low ⨁⨁◯◯
Postoperative IVH	3/431	4.36 (1.16–16.44)	Serious	Serious	Not serious	Serious	Not assessed †	Large	Very low ⨁◯◯◯
Postoperative CSF leakage	4/404	3.07 (0.55–17.07)	Serious	Serious	Not serious	Very serious	Not assessed †	None ¶	Very low ⨁◯◯◯
Postoperative infection	5/552 #	4.96 (1.77–13.91)	Serious	Not serious **	Not serious	Serious	Not assessed †	Large	Low ⨁⨁◯◯
Pseudomeningocele	3/229	6.17 (3.12–12.19)	Serious	Not serious	Not serious	Not serious	Not assessed †	Large	Low ⨁⨁◯◯

Note: CI, confidence interval; CSF, cerebrospinal fluid; EVD, external ventricular drain; IVH, intraventricular hemorrhage; OR, odds ratio; VPS, ventriculoperitoneal shunt. Certainty was assessed using the modified GRADE framework for prognostic-factor research, with predominantly phase-1 evidence starting at moderate certainty. All pooled estimates were derived from crude/unadjusted data; therefore, * magnitude of association was reported descriptively and did not result in upgrading of certainty because residual confounding could not be excluded. † Publication bias and small-study effects could not be reliably assessed because fewer than 10 studies contributed to each factor-specific analysis. ‡ EVD was considered particularly susceptible to confounding by indication. § For the inverse association with astrocytoma, magnitude was considered using the reciprocal OR. ¶ No magnitude designation was applied to CSF leakage because the confidence interval crossed the null and was extremely wide. # Five unique studies contributed six comparisons to the postoperative infection analysis. ** Overall heterogeneity for postoperative infection was moderate (I^2^ = 49.1%) and was not considered sufficient to warrant downgrading, although greater heterogeneity was observed within the CSF-infection subgroup.

## Data Availability

No new data were created or analyzed in this study. Data sharing is not applicable to this article.

## Supplementary Materials

The following supporting information can be downloaded at: https://www.mdpi.com/article/10.3390/medicina62101953/s1, Table S1: Statistical adjustment, confounding control, and adjusted effect estimates in the included studies; Table S2: Definitions and assessment of hydrocephalus, temporary CSF diversion, and indications for postoperative VP shunt placement; Table S3: Domain-level risk-of-bias assessment using the QUIPS tool; Table S4: Narrative synthesis of additional tumor-related characteristics according to postoperative VP shunt placement following PFT resection; Figure S1. Leave-One-Out Sensitivity Analysis for Pseudomeningocele and VP Shunt Placement; Figure S2. Leave-One-Out Sensitivity Analysis for Ependymoma and VP Shunt Placement; Figure S3. Leave-One-Out Sensitivity Analysis for Midline Tumor Location and VP Shunt Placement; Figure S4. Leave-One-Out Sensitivity Analysis for Postoperative CSF Leakage and VP Shunt Placement; Figure S5. Leave-One-Out Sensitivity Analysis for Medulloblastoma and VP Shunt Placement; Figure S6. Leave-One-Out Sensitivity Analysis for Astrocytoma and VP Shunt Placement; Figure S7. Temporal Subgroup Analysis of EVD Exposure and VP Shunt Placement; Figure S8. Temporal Subgroup Analysis of Subtotal Resection and VP Shunt Placement; Figures S9–S16: Factor-specific sensitivity analyses after exclusion of historical cohorts; Figure S9. Midline tumor location; Figure S10. Medulloblastoma; Figure S11. Astrocytoma; Figure S12. Ependymoma; Figure S13. EVD exposure; Figure S14. EVD exposure by subgroup; Figure S15. Incomplete tumor resection; Figure S16. Postoperative infection; File S1. PRISMA checklist [32].

## Abbreviations

The following abbreviations are used in this manuscript:

CI	Confidence Interval
CSF	Cerebrospinal Fluid
ETV	Endoscopic Third Ventriculostomy
EVD	External Ventricular Drain
GRADE	Grading of Recommendations Assessment, Development and Evaluation
GTR	Gross Total Resection
HC	Hydrocephalus
IVH	Intraventricular Hemorrhage
MB	Medulloblastoma
OR	Odds Ratio
PFT	Posterior Fossa Tumor
PMC	Pseudomeningocele
PRISMA	Preferred Reporting Items for Systematic Reviews and Meta-Analyses
PROSPERO	International Prospective Register of Systematic Reviews
QUIPS	Quality In Prognosis Studies
STR	Subtotal Resection
VP Shunt	Ventriculoperitoneal Shunt
