# Supplementary material for: Factors Associated with Ventriculoperitoneal Shunt Placement Following Posterior Fossa Tumor Resection in Children: A Systematic Review and Meta-Analysis
Source: Medicina (Kaunas). 2026 Oct 9;62(10):1953. doi: 10.3390/medicina62101953 (PMC13706951; doi:10.3390/medicina62101953)
Supplement: Supplementary file 1 [file medicina-62-01953-s001.zip › File S1. Prisma checklist.pdf]

PRISMA 2020 Checklist

Section and Topic	Item #	Checklist item	Location where item is reported
**TITLE**			
Title	1	Identify the report as a systematic review.	Title.
**ABSTRACT**			
Abstract	2	See the PRISMA 2020 for Abstracts checklist.	Abstract (structured summary; see PRISMA for Abstracts for item-level assessment).
**INTRODUCTION**			
Rationale	3	Describe the rationale for the review in the context of existing knowledge.	Introduction, paragraphs 1–4.
Objectives	4	Provide an explicit statement of the objective(s) or question(s) the review addresses.	Introduction, final paragraph.
**METHODS**			
Eligibility criteria	5	Specify the inclusion and exclusion criteria for the review and how studies were grouped for the syntheses.	Materials and Methods, “Eligibility Criteria and Study Selection”; “Data Extraction and Outcomes Measures” (factor grouping).
Information sources	6	Specify all databases, registers, websites, organisations, reference lists and other sources searched or consulted to identify studies. Specify the date when each source was last searched or consulted.	Materials and Methods, “Study Design and Literature Search” (six sources and reference lists). Search date by source is not stated; Abstract says through April 2025.
Search strategy	7	Present the full search strategies for all databases, registers and websites, including any filters and limits used.	Materials and Methods, “Study Design and Literature Search” (example terms and limits). Full database-specific strategies are not reported.
Selection process	8	Specify the methods used to decide whether a study met the inclusion criteria of the review, including how many reviewers screened each record and each report retrieved, whether they worked independently, and if applicable, details of automation tools used in the process.	Materials and Methods, “Eligibility Criteria and Study Selection” (two independent reviewers and consensus).
Data collection process	9	Specify the methods used to collect data from reports, including how many reviewers collected data from each report, whether they worked independently, any processes for obtaining or confirming data from study investigators, and if applicable, details of automation tools used in the process.	Materials and Methods, “Data Extraction and Outcomes Measures” (two independent reviewers and standard form). Investigator-contact process is not stated.
Data items	10a	List and define all outcomes for which data were sought. Specify whether all results that were compatible with each outcome domain in each study were sought (e.g. for all measures, time points, analyses), and if not, the methods used to decide which results to collect.	Materials and Methods, “Data Extraction and Outcomes Measures” (primary outcome and factor categories). Selection among multiple eligible time points or estimates is not fully specified.
	10b	List and define all other variables for which data were sought (e.g. participant and intervention characteristics, funding sources). Describe any assumptions made about any missing or unclear information.	Materials and Methods, “Data Extraction and Outcomes Measures”; Supplementary Tables S1–S2. Handling of missing or unclear data is not fully described.
Study risk of bias assessment	11	Specify the methods used to assess risk of bias in the included studies, including details of the tool(s) used, how many reviewers assessed each study and whether they worked independently, and if applicable, details of automation tools used in the process.	Materials and Methods, “Risk-of-Bias Assessment”; Supplementary Table S3.
Effect measures	12	Specify for each outcome the effect measure(s) (e.g. risk ratio, mean difference) used in the synthesis or presentation of results.	Materials and Methods, “Statistical Analysis” (odds ratios and 95% confidence intervals).
Synthesis methods	13a	Describe the processes used to decide which studies were eligible for each synthesis (e.g. tabulating the study intervention characteristics and comparing against the planned groups for each synthesis (item #5)).	Materials and Methods, “Eligibility Criteria and Study Selection”; “Data Extraction and Outcomes Measures”; “Statistical Analysis” (at least two studies per factor).
	13b	Describe any methods required to prepare the data for presentation or synthesis, such as handling of missing summary statistics, or data conversions.	Materials and Methods, “Data Extraction and Outcomes Measures” (incident postoperative events and crude versus adjusted data). Data-conversion methods are not stated.
	13c	Describe any methods used to tabulate or visually display results of individual studies and syntheses.	Materials and Methods, “Statistical Analysis”; Results, Table 1, Figures 2–7, Table 2 and Supplementary Tables S1–S4.

PRISMA

PRISMA 2020 Checklist

Section and Topic	Item #	Checklist item	Location where item is reported
	13d	Describe any methods used to synthesize results and provide a rationale for the choice(s). If meta-analysis was performed, describe the model(s), method(s) to identify the presence and extent of statistical heterogeneity, and software package(s) used.	Materials and Methods, "Statistical Analysis" (random effects, Q, I^2, R 4.5.1, meta package).
	13e	Describe any methods used to explore possible causes of heterogeneity among study results (e.g. subgroup analysis, meta-regression).	Materials and Methods, "Statistical Analysis" (infection and EVD subgroups); Results, Figures 4 and 7.
	13f	Describe any sensitivity analyses conducted to assess robustness of the synthesized results.	Materials and Methods, "Statistical Analysis" (leave-one-out and historical-cohort exclusions).
Reporting bias assessment	14	Describe any methods used to assess risk of bias due to missing results in a synthesis (arising from reporting biases).	Materials and Methods, "Statistical Analysis" (no formal funnel-plot/asymmetry test because each factor had fewer than 10 studies).
Certainty assessment	15	Describe any methods used to assess certainty (or confidence) in the body of evidence for an outcome.	Materials and Methods, "Certainty of Evidence" (modified GRADE for prognostic factors).
**RESULTS**			
Study selection	16a	Describe the results of the search and selection process, from the number of records identified in the search to the number of studies included in the review, ideally using a flow diagram.	Results, "Study selection"; Figure 1.
	16b	Cite studies that might appear to meet the inclusion criteria, but which were excluded, and explain why they were excluded.	Results, "Study selection" and Figure 1 (aggregate exclusion reasons). Individual potentially eligible excluded reports are not cited with reasons.
Study characteristics	17	Cite each included study and present its characteristics.	Results, "Study characteristics"; Table 1; Supplementary Tables S1–S2.
Risk of bias in studies	18	Present assessments of risk of bias for each included study.	Results, risk-of-bias paragraph and Figure 2; Supplementary Table S3 (domain-level judgments).
Results of individual studies	19	For all outcomes, present, for each study: (a) summary statistics for each group (where appropriate) and (b) an effect estimate and its precision (e.g. confidence/credible interval), ideally using structured tables or plots.	Results, Figures 3–7 (study-specific estimates and confidence intervals). Group-level event counts should be checked in the plots; no separate per-study data table is cited.
Results of syntheses	20a	For each synthesis, briefly summarise the characteristics and risk of bias among contributing studies.	Results, "Study characteristics", Table 1, Figure 2 and factor-specific Results (study numbers); study-specific bias in each synthesis is not explicitly tabulated.
	20b	Present results of all statistical syntheses conducted. If meta-analysis was done, present for each the summary estimate and its precision (e.g. confidence/credible interval) and measures of statistical heterogeneity. If comparing groups, describe the direction of the effect.	Results, factor-specific paragraphs and Figures 3–7; Table 2 (pooled ORs, CIs, heterogeneity in text).
	20c	Present results of all investigations of possible causes of heterogeneity among study results.	Results, EVD and infection subgroup findings (Figures 4 and 7); historical-cohort analyses (Supplementary Figures S9–S16).
	20d	Present results of all sensitivity analyses conducted to assess the robustness of the synthesized results.	Results, sensitivity-analysis paragraph; Supplementary Figures S1–S16.
Reporting biases	21	Present assessments of risk of bias due to missing results (arising from reporting biases) for each synthesis assessed.	Results, Table 2 (publication bias marked "Not assessed"); Methods, "Statistical Analysis" (reason). No formal synthesis-level assessment performed.
Certainty of evidence	22	Present assessments of certainty (or confidence) in the body of evidence for each outcome assessed.	Results, certainty-of-evidence paragraph and Table 2.
**DISCUSSION**			
Discussion	23a	Provide a general interpretation of the results in the context of other evidence.	Discussion, opening and factor-specific interpretation.
	23b	Discuss any limitations of the evidence included in the review.	Discussion, "Several limitations should be acknowledged" paragraph.

PRISMA 2020 Checklist

Section and Topic	Item #	Checklist item	Location where item is reported
	23c	Discuss any limitations of the review processes used.	Discussion, limitations paragraph (crude pooling, inconsistent reporting, heterogeneity); review-process limitations are not clearly separated.
	23d	Discuss implications of the results for practice, policy, and future research.	Discussion, "Clinical Implications" and final future-research paragraph; Conclusions.
**OTHER INFORMATION**			
Registration and protocol	24a	Provide registration information for the review, including register name and registration number, or state that the review was not registered.	Materials and Methods, "Study Design and Literature Search"; Abstract (PROSPERO CRD420261455396).
	24b	Indicate where the review protocol can be accessed, or state that a protocol was not prepared.	Materials and Methods, "Study Design and Literature Search" (PROSPERO registration). Direct protocol-access location is not provided.
	24c	Describe and explain any amendments to information provided at registration or in the protocol.	Not reported: amendments to registration/protocol, or an explicit statement that none occurred.
Support	25	Describe sources of financial or non-financial support for the review, and the role of the funders or sponsors in the review.	Funding ("no external funding"); Acknowledgments (institutional support). Role of any nonfinancial supporters is not specified.
Competing interests	26	Declare any competing interests of review authors.	Conflicts of Interest.
Availability of data, code and other materials	27	Report which of the following are publicly available and where they can be found: template data collection forms; data extracted from included studies; data used for all analyses; analytic code; any other materials used in the review.	Data Availability Statement; Supplementary Materials (Tables S1–S4 and Figures S1–S16). Extracted meta-analysis data available on request; public availability of forms and analytic code is not stated.

From: Page MJ, McKenzie JE, Bossuyt PM, Boutron I, Hoffmann TC, Mulrow CD, et al. The PRISMA 2020 statement: an updated guideline for reporting systematic reviews. BMJ 2021;372:n71. doi: 10.1136/bmj.n71.
This work is licensed under CC BY 4.0. To view a copy of this license, visit https://creativecommons.org/licenses/by/4.0/
