# Supplementary material for: Factors Associated with Ventriculoperitoneal Shunt Placement Following Posterior Fossa Tumor Resection in Children: A Systematic Review and Meta-Analysis
Source: Medicina (Kaunas). 2026 Oct 9;62(10):1953. doi: 10.3390/medicina62101953 (PMC13706951; doi:10.3390/medicina62101953)
Supplement: Supplementary file 1 [file medicina-62-01953-s001.zip › Tables S1-S4 and Figures S1-S16.pdf]

Supplementary Table S1. Statistical adjustment, confounding control, and adjusted effect estimates in the included studies

Study	Univariable analysis	Multivariable / confounder-adjusted analysis	Variables/covariates included or controlled	Principal adjusted findings	Classification for present review
**Abraham 2019**	Yes	**Yes — penalized logistic regression**	Age, tumor location, EVD, extent of resection, meningitis, tumor aggressiveness/pathology, postoperative intraventricular blood	Age ≤6 y: **aOR 5.9 (1.6–22.6)**; IVB: **aOR 6.4 (1.7–23.7)**. Midline location 2.9 (0.4–20.3); PR 2.7 (0.6–12.1); meningitis 3.7 (0.7–20.2); aggressive tumor 0.7 (0.2–3.1)	**Fully adjusted multivariable study**
**Afshari 2023**	Yes	**Yes — stepwise multivariable logistic regression**	Molecular subgroup, leptomeningeal/metastatic disease, preoperative HC, resection status, sex	Preop HC: **aOR 8.245 (1.306–52.069)**; leptomeningeal disease: **7.076 (1.426–35.118)**; molecular subgroup 3.154 (0.576–17.258); resection 0.526 (0.085–3.241); sex 2.790 (0.538–14.479)	**Fully adjusted multivariable study**
**Bhuyan 2020**	Yes	**No**	None	Individual comparisons only	**Unadjusted**
**Culley 1994**	Yes	**Partial adjustment — stratified Mantel–Haenszel analyses**	Age (<3/≥3 y) and tumor location used as confounders for selected surgical/postoperative factors	Adjustment particularly used to assess dural graft and tumor-type associations; several crude associations weakened after controlling for age/location	**Partially adjusted**
**Dias & Albright 1989**	Yes	**Yes — Cox proportional-hazards model**	Multiple clinical, radiological and pathological variables entered simultaneously	Smaller extent of tumor resection and open dura independently associated with subsequent shunting; reported estimates not directly equivalent to conventional logistic ORs	**Multivariable time-to-event study**
**Helmbold 2019**	Yes	**No**	None; ROC used to derive FOHR and CRP thresholds	Reported factor-specific unadjusted associations	**Unadjusted**
**Hu 2023**	Yes	**Yes — stepwise forward multivariable logistic regression**	Significant univariable variables plus tumor-related variables, including resection, pathology, recurrence, cavity blood clot and tumor size	Age <3 y: **aOR 3.760 (1.294–10.927)**; blood loss: **1.601 (1.196–2.143)**; fourth-ventricle location: **7.697 (2.595–22.835)**	**Fully adjusted multivariable study**
**Kumar 1996**	Yes	**Partial adjustment — Mantel–Haenszel**	Confounding variable controlled where appropriate; full multivariable covariate set not reported	Primarily group comparisons; no consistent multivariable-adjusted OR series	**Partially adjusted**
**Lee 1994**	Yes	**No formal multivariable model**	None; authors evaluated dependency/interrelationship among variables qualitatively	Age, hydrocephalus severity and tumor stage associated with shunting; resection effect depended on tumor stage	**Unadjusted**

Note: aOR, adjusted odds ratio; **EVD**, external ventricular drainage; **HC**, hydrocephalus; **IVB**, intraventricular blood; **PR**, partial resection. The factor-specific meta-analyses in the present review were predominantly based on crude event data. An adjusted-only meta-analysis was not undertaken because sufficiently comparable adjusted estimates were generally unavailable for the same exposure, predictor definitions differed between studies, covariate sets were heterogeneous, and Dias and Albright used a time-to-event model rather than logistic regression.

Supplementary Table S2. Definitions and assessment of hydrocephalus, temporary CSF diversion, and indications for postoperative VP shunt placement

Study	Preoperative HC definition / assessment	Postoperative HC assessment	EVD strategy / definition	Specific indication for postoperative VPS	Timing definition
**Abraham 2019**	Evans ratio on CT/MRI; **>0.30 = ventriculomegaly**	Clinical symptomatic HC + imaging; early postoperative period defined as ≤30 d	Preop EVD only for persistent bradycardia or altered sensorium; 10 patients received preop EVD; 3 additional postop EVD	Persistent raised ICP symptoms (6/14); wound CSF leak (7/14); tense pseudomeningocele (1/14)	Early VPS ≤30 d; median 14 d
**Afshari 2023**	Presence of HC on preoperative imaging; no quantitative ventricular threshold stated	Persistent/new HC requiring CSF diversion	Preoperative EVD recorded as a clinical variable	Specific standardized shunt threshold NR; postoperative VPS requirement obtained from records	Median 0.5 mo, range 0.13–27.6 mo
**Bhuyan 2020**	HC evaluated using temporal horn size, Evans ratio, FH:IPD ratio and periventricular lucency	Clinical/radiological postoperative HC	Postoperative EVD insertion and duration recorded	VPS when patient developed **EVD dependence, hydrocephalus, or pseudomeningocele**	NR
**Culley 1994**	Presence/absence based on radiology report; severity classified as mild/moderate/marked where available	Chart/imaging evidence; VPS postoperative day documented	Symptomatic intracranial hypertension treated with EVD before or preferably at surgery; EVD presence and duration assessed	Hydrocephalus; EVD dependence; persistent pseudomeningocele; mental-status changes; CSF leak; tumor recurrence with obstruction	69% within 30 d
**Dias & Albright 1989**	HC graded **mild/moderate/severe** on CT/MRI; clinical HC grade also assigned	Serial clinical assessment and follow-up CT; transient symptoms distinguished from persistent HC	17 initially managed with perioperative EVD; 16 received no initial CSF diversion	Persistent/recurrent symptomatic HC after EVD removal or conservative management	Time-to-shunt incorporated into Cox model; mean follow-up ~52 mo
**Helmbold 2019**	Radiological HC on first preoperative MRI; FOHR calculated; FOHR ≤0.37 described as physiologic reference	MRI within 72 h; postoperative HC, transependymal edema and FOHR quantified; **FOHR >0.46** identified as high-risk threshold	Pre-, intra-, and postoperative EVD documented; 22/70 received perioperative EVD	Clinical **and radiological signs of persistent HC**; explicit standardized threshold beyond this NR	Mean 16.6 d; median 16 d, range 7–27 d
**Hu 2023**	MRI + symptoms + **Evans ratio >0.30**	New postoperative HC or progression of preoperative HC	Preoperative EVD recorded	(1) Postoperative-onset HC or progression of preop HC **and** (2) failure of conservative treatment	Most progression within 2 weeks; late VPS also reported
**Kumar 1996**	Hydrocephalus documented on preoperative CT/MRI; quantitative threshold NR	Persistent postoperative HC and clinical postoperative complications	Preop EVD or ventricular reservoir used in moribund/persistently symptomatic patients despite steroids	Persistent HC; CSF leak or pseudomeningocele failing serial lumbar punctures/lumbar drainage	Majority within 45 d
**Lee 1994**	Quantified using **frontal horn ratio** and **ventricular body ratio**; severity of preoperative HC assessed	Persistent abnormal CSF circulation with symptoms/signs after tumor resection	Perioperative EVD recorded	Continued HC with raised ICP, wound pseudomeningocele and/or wound CSF leak; primary endpoint permanent shunt within 4 weeks	Primary VPS outcome ≤4 weeks; two later recurrence-related shunts

Supplementary Table S3. Domain-Level Risk-of-Bias Assessment Using the QUIPS Tool

Study	Study Participation	Study Attrition	Prognostic Factor Measurement	Outcome Measurement	Study Confounding	Statistical Analysis & Reporting	Overall Risk of Bias
**Abraham et al., 2019**	Low	Low	Low	Low	Moderate	Low	**Low**
**Afshari et al., 2023**	Moderate	Low	Moderate	Low	Moderate	High	**High**
**Bhuyan et al., 2020**	Moderate	Moderate	Low	Low	Moderate	Moderate	**Moderate**
**Culley et al., 1994**	Moderate	Moderate	Moderate	Low	High	Moderate	**High**
**Dias & Albright, 1989**	Moderate	Moderate	Moderate	Low	High	Moderate	**High**
**Helmbold et al., 2019**	Low	Low	Low	Low	Moderate	Low	**Low**
**Hu et al., 2023**	Low	Low	Low	Low	Low	Low	**Low**
**Kumar et al., 1996**	Moderate	Moderate	Moderate	Low	High	Moderate	**High**
**Lee et al., 1994**	Moderate	Moderate	Moderate	Low	Moderate	Moderate	**Moderate**

Supplementary Table S4. Narrative synthesis of additional tumor-related characteristics according to postoperative VP shunt placement following PFT resection

Variable	Study	Category / definition	Postoperative VP shunt, n/N (%)	No postoperative VP shunt, n/N (%)	Association with postoperative VP shunt placement
Tumor size	Hu et al., 2023	>30 mm	23/29 (79.3%)	125/188 (66.5%)	OR 1.93; p=0.173
		≤30 mm	6/29 (20.7%)	63/188 (33.5%)	Reference
Tumor grade / pathological classification	Hu et al., 2023	WHO grade III–IV	22/29 (75.9%)	95/188 (50.5%)	OR 3.08; p=0.014
		WHO grade I–II	7/29 (24.1%)	93/188 (49.5%)	Reference
	Abraham et al., 2019	Aggressive pathology*	11/14 (78.6%)	82/134 (61.2%)	Crude OR 2.30 (95% CI 0.60–8.73), p=0.21; aOR 0.70 (95% CI 0.20–3.10), p=0.60
		Benign pathology*	3/14 (21.4%)	52/134 (38.8%)	Reference
Molecular subgroup	Afshari et al., 2023	WNT	0/10 (0%)	10/10 (100%)	—
		SHH	1/12 (8.3%)	11/12 (91.7%)	—
		Group 3	7/19 (36.8%)	12/19 (63.2%)	—
		Group 4	5/15 (33.3%)	10/15 (66.7%)	—
		Group 3/4, unspecified	3/13 (23.1%)	10/13 (76.9%)	—
		Overall molecular comparison	—	—	Univariable p=0.0394; aOR 3.15 (95% CI 0.58–17.26), p=0.185
Fourth ventricular anatomy/location	Hu et al., 2023	Fourth-ventricular tumor	23/29 (79.3%)	62/188 (33.0%)	Crude OR 7.79, p<0.001; aOR 7.70 (95% CI 2.60–22.84), p<0.001
		Other location	6/29 (20.7%)	126/188 (67.0%)	Reference
	Culley et al., 1994	Free CSF flow through fourth ventricle after tumor resection	15/48 (31.3%)	33/48 (68.8%)	Descriptive only; fourth-ventricular CSF-flow status was not documented for the remaining 69 patients

Note: The outcome was postoperative VP shunt placement following PFT resection; preoperative shunting, ETV, and hydrocephalus without subsequent VP shunting were not considered outcome events. Only studies with extractable quantitative data are presented. Owing to heterogeneous definitions and categorizations, these variables were synthesized narratively rather than pooled. *Abraham et al. classified pilocytic astrocytoma and choroid plexus papilloma as benign and medulloblastoma, ependymoma, and high-grade glioma as aggressive; 14/148 patients required postoperative VP shunting.

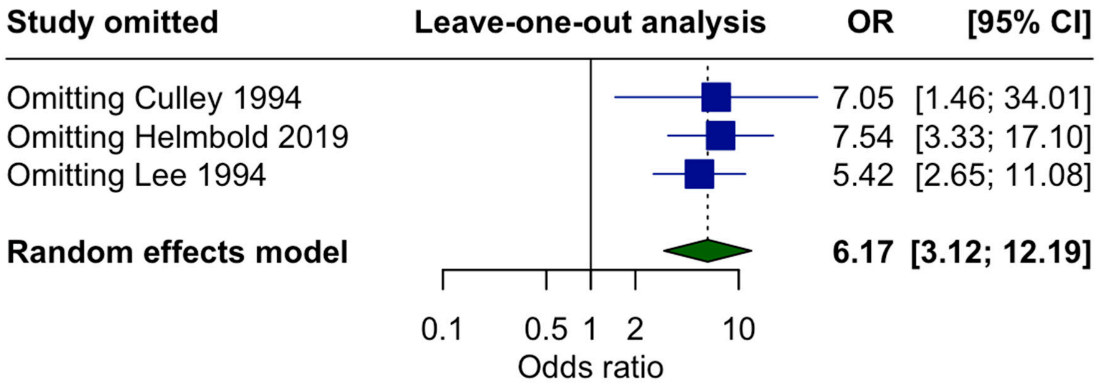

Figure S1. Leave-One-Out Sensitivity Analysis for Pseudomeningocele and VP Shunt Placement

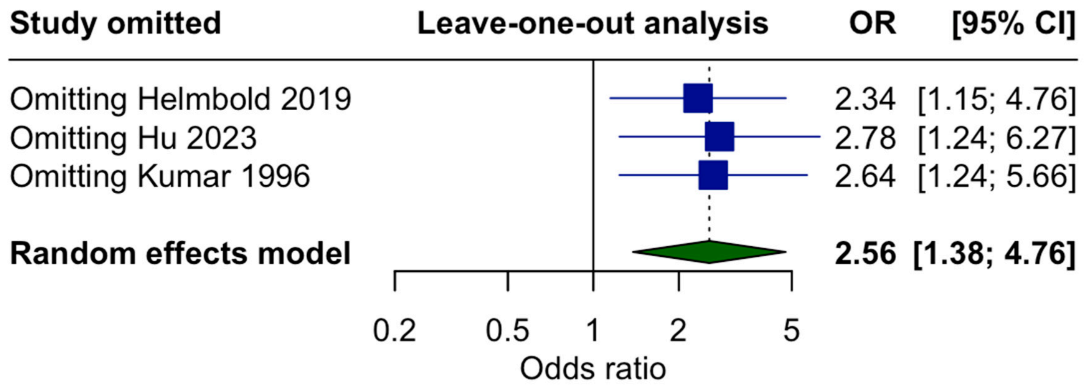

Figure S2. Leave-One-Out Sensitivity Analysis for Ependymoma and VP Shunt Placement

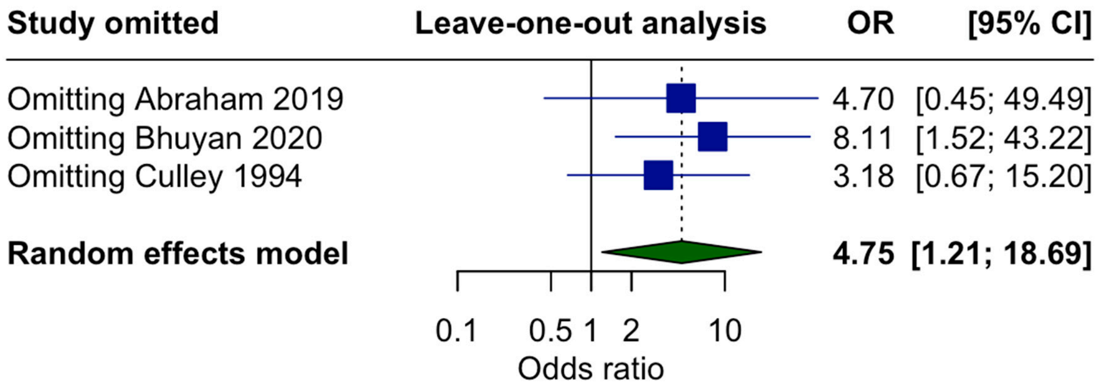

Figure S3. Leave-One-Out Sensitivity Analysis for Midline Tumor Location and VP Shunt Placement

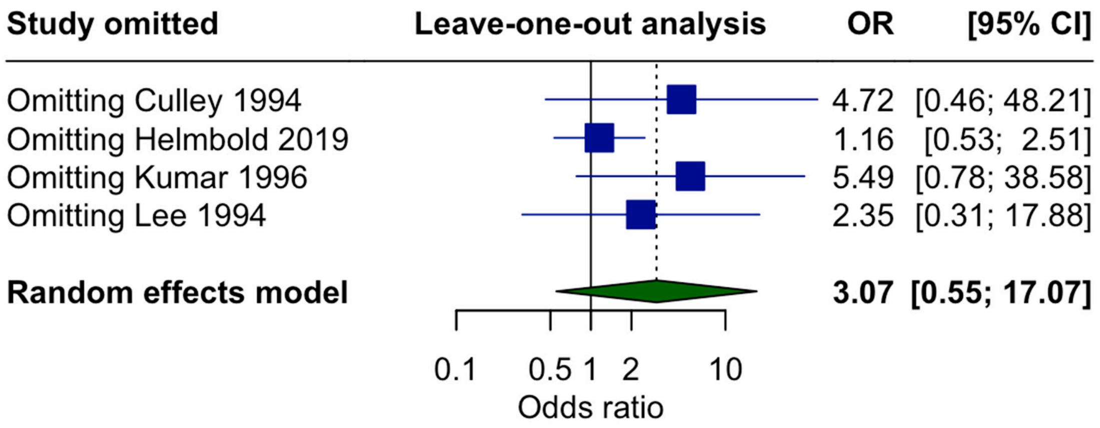

Figure S4. Leave-One-Out Sensitivity Analysis for Postoperative CSF Leakage and VP Shunt Placement

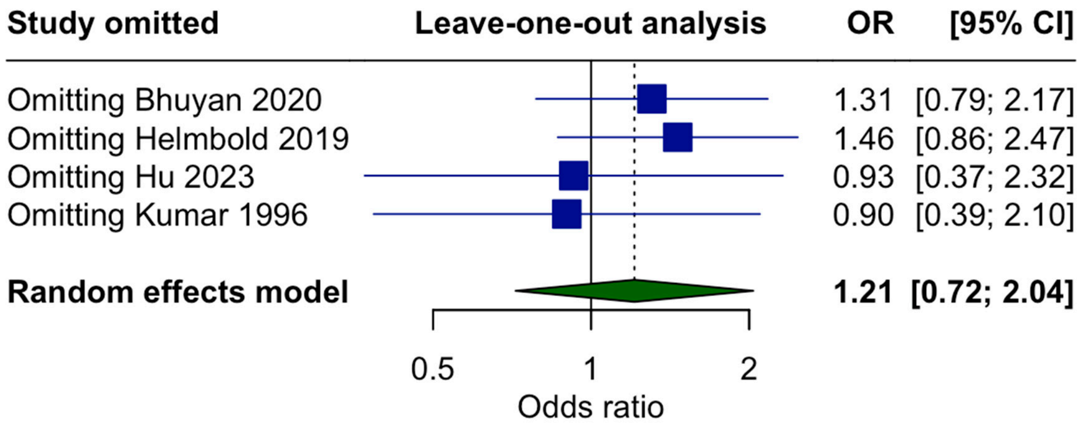

Figure S5. Leave-One-Out Sensitivity Analysis for Medulloblastoma and VP Shunt Placement

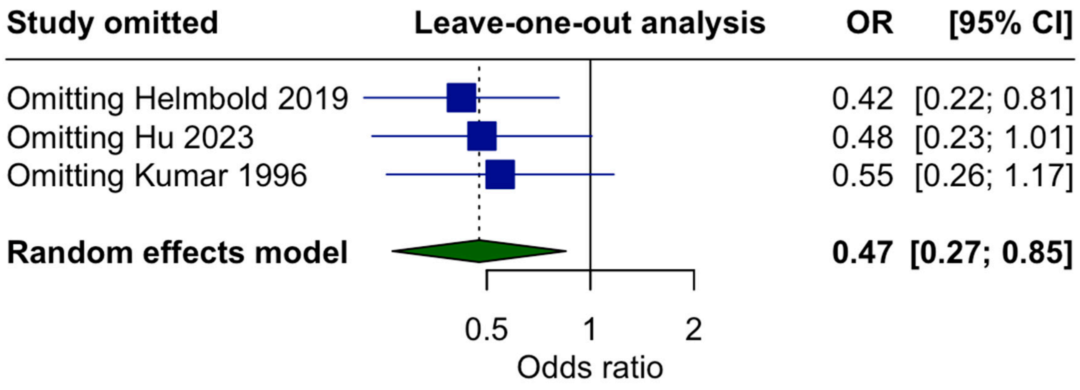

Figure S6. Leave-One-Out Sensitivity Analysis for Astrocytoma and VP Shunt Placement

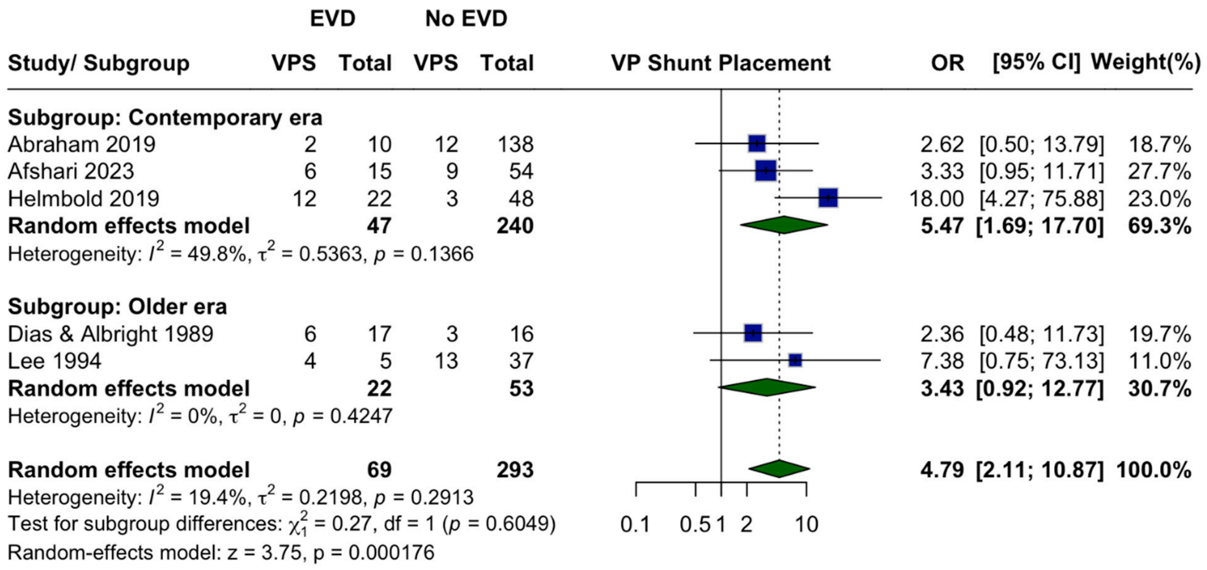

Figure S7. Temporal Subgroup Analysis of EVD Exposure and VP Shunt Placement

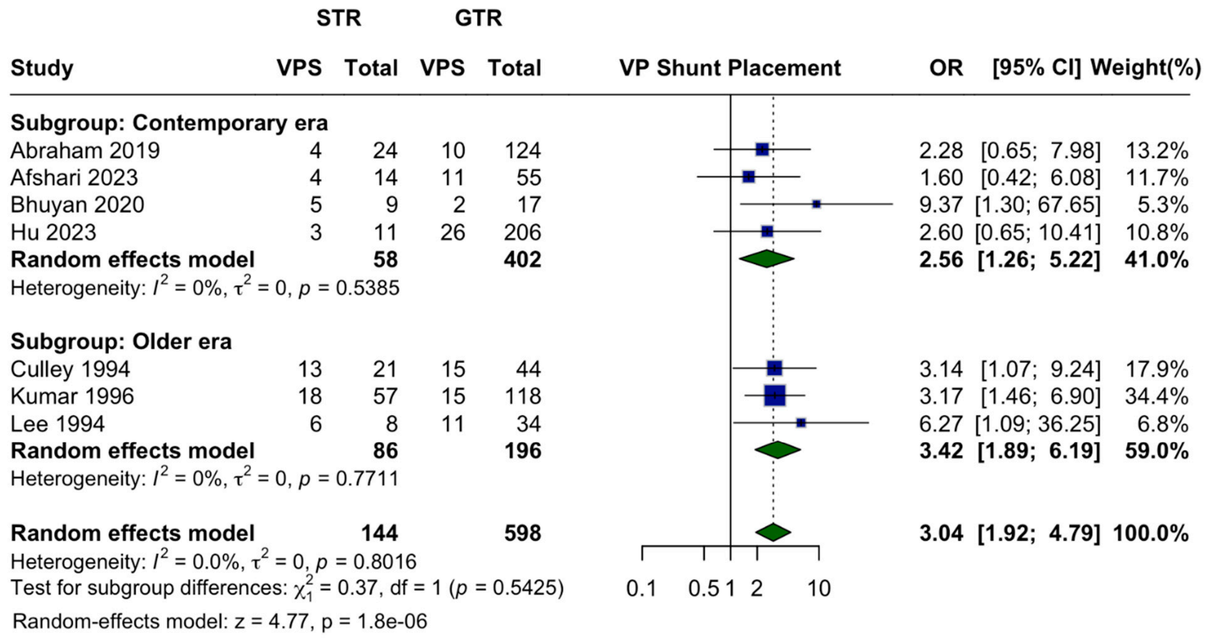

Figure S8. Temporal Subgroup Analysis of Subtotal Resection and VP Shunt Placement

Factor-Specific Sensitivity Analyses Excluding Historical Cohorts (1994–1996):

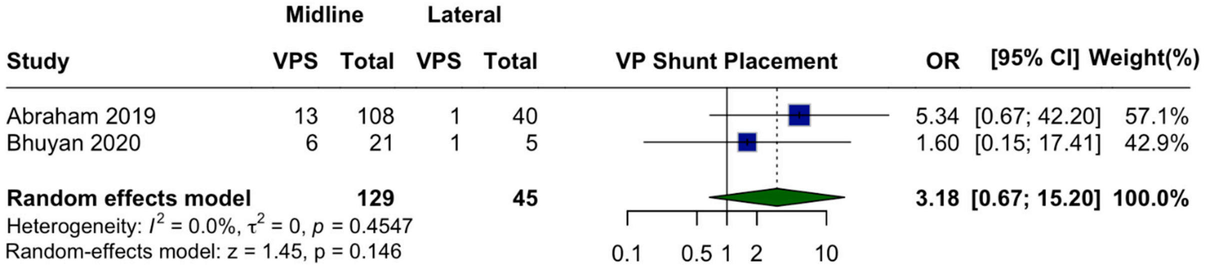

Figure S9. **Midline tumor location**. Forest plot of the association between midline tumor location and VP shunt placement after exclusion of the historical cohort. The association was attenuated and no longer statistically significant (OR 3.18, 95% CI 0.67–15.20; I^2 = 0%).

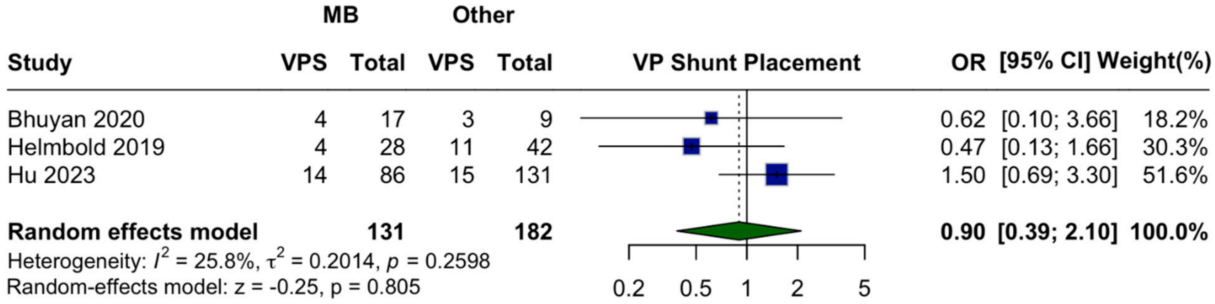

Figure S10. Medulloblastoma. Forest plot of the association between medulloblastoma histology and VP shunt placement after exclusion of the historical cohort. No significant association was observed (OR 0.90, 95% CI 0.39–2.10; I^2 = 25.8%).

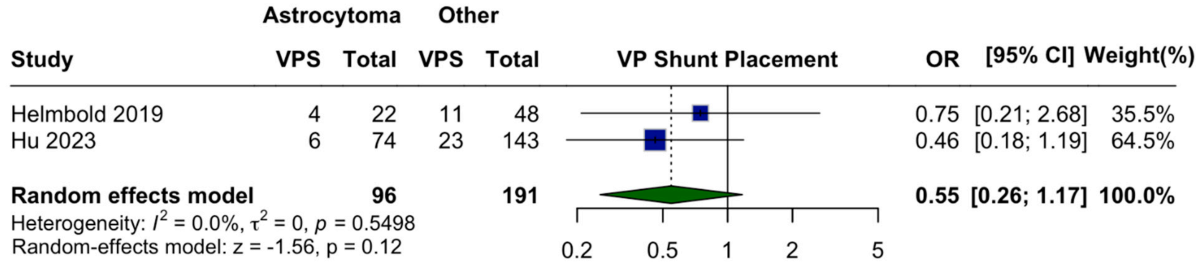

Figure S11. Astrocytoma. Forest plot of the association between astrocytoma histology and VP shunt placement after exclusion of the historical cohort. The association was attenuated and no longer statistically significant (OR 0.55, 95% CI 0.26–1.17; I^2 = 0%).

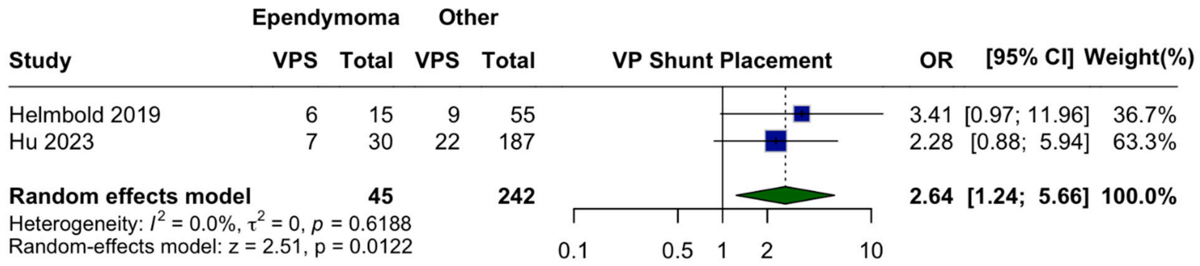

Figure S12. Ependymoma. Forest plot of the association between ependymoma histology and VP shunt placement after exclusion of the historical cohort. Ependymoma remained associated with higher odds of VP shunt placement (OR 2.64, 95% CI 1.24–5.66; I^2 = 0%).

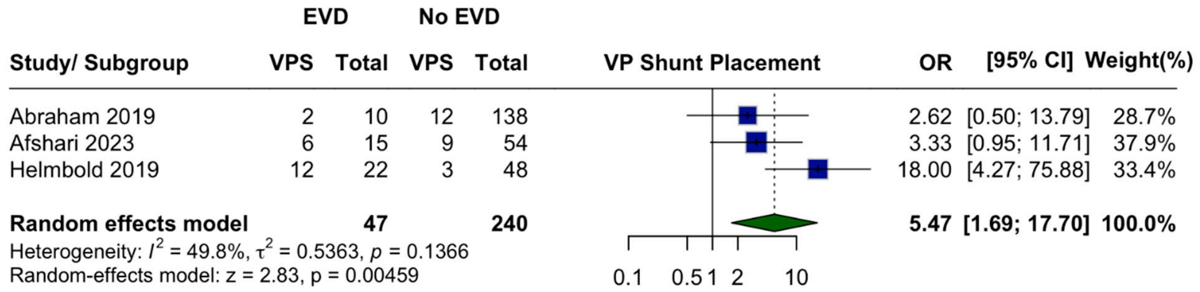

Figure S13. EVD exposure. Forest plot of the association between EVD exposure and subsequent VP shunt placement after exclusion of the historical cohort. EVD exposure remained associated with higher odds of VP shunt placement (OR 5.47, 95% CI 1.69–17.70; I^2 = 49.8%).

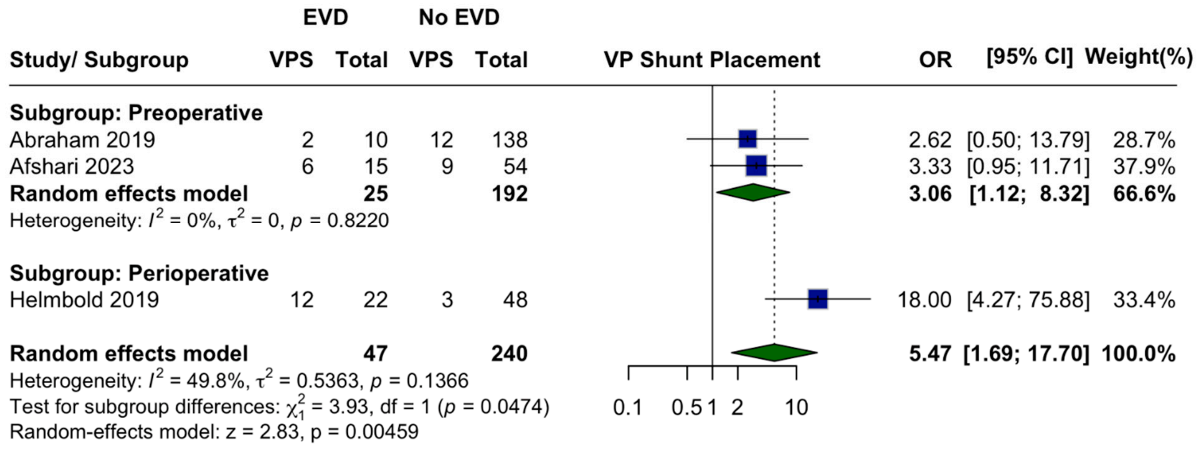

Figure S14. EVD exposure by subgroup. Forest plot of the association between EVD exposure and subsequent VP shunt placement stratified by preoperative and perioperative timing after exclusion of the historical cohort. The overall association remained significant (OR 5.47, 95% CI 1.69–17.70; I^2 = 49.8%). Subgroup findings should be interpreted cautiously because the perioperative subgroup was represented by a single study.

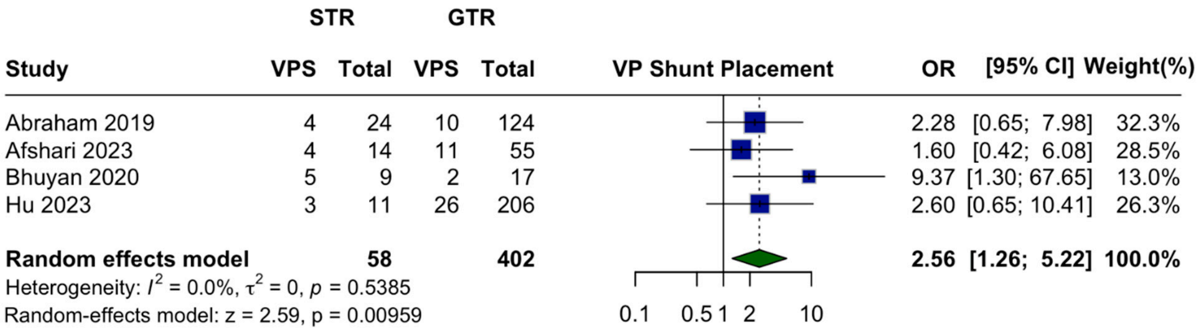

Figure S15. Incomplete tumor resection. Forest plot of the association between incomplete tumor resection and VP shunt placement after exclusion of historical cohorts. Incomplete tumor resection remained associated with higher odds of VP shunt placement (OR 2.56, 95% CI 1.26–5.22; I^2 = 0%).

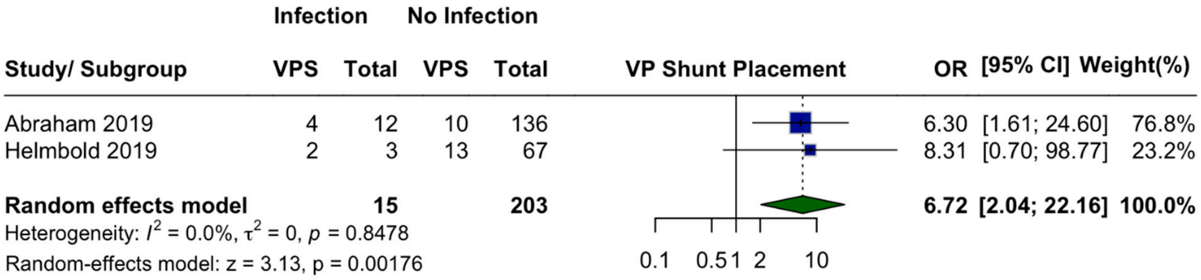

Figure S16. Postoperative infection. Forest plot of the association between postoperative infection and VP shunt placement after exclusion of the historical cohort. Postoperative infection remained associated with higher odds of VP shunt placement (OR 6.72, 95% CI 2.04–22.16; I^2 = 0%).
